# Supplementary material for: ClinASO: An open-source platform for rapid drug discovery of gapmer antisense oligonucleotides
Source: Mol Ther Nucleic Acids. 2026 Apr 16;37(2):102933. doi: 10.1016/j.omtn.2026.102933 (PMC13156736; doi:10.1016/j.omtn.2026.102933)
Supplement: Document S1. Figures S1–S5 and Table S2 [file mmc1.pdf]

OMTN, Volume *37*

Supplemental information

ClinASO: An open-source platform for rapid drug discovery of gapmer antisense oligonucleotides

Shunkai Chen, Hao Liu, Dezi Cong, Ao Dong, Yuhang Wang, Jingyi Bi, Shijie Guo, Juan Yang, Xiaolei Wang, Guiping Ren, Ke Zhang, Haisheng Wang, Fan Lai, and Yunkun Dang

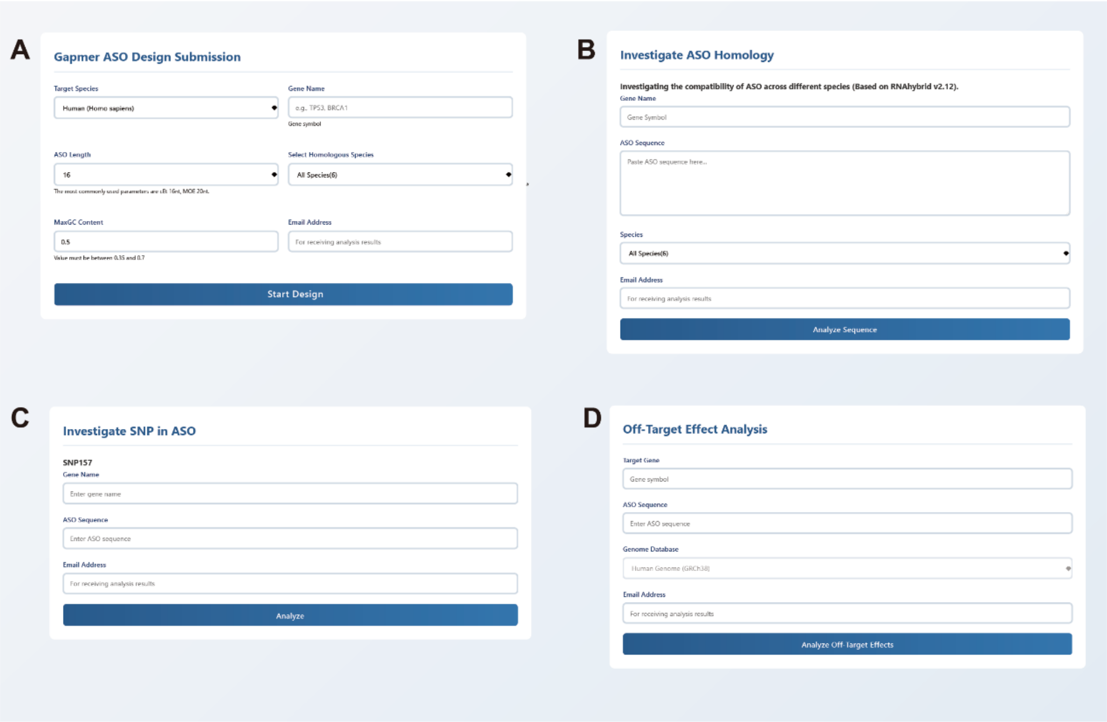

Figure S1. Overview of the four main functional interfaces of the ClinASO platform.

(A) Gapmer ASO design interface.

(B) ASO homology analysis interface.

(C) SNP Investigate tool for ASOs.

(D) Off-target prediction interface

A

Drug Name	Trade Name	Developer	Approval Year	Target
Fomivirsen	Vitravene	Ionis Pharmaceuticals/Novartis	1998	CMV
Mipomersen	Kynamro	Ionis/Sanofi	2013	ApoB-100
Inotersen	Tegsedi	Ionis/Akcea Therapeutics	2018	TTR
Tofersen	Qalsody	Ionis/Biogen	2023	SOD1
Eplontersen	Wainua	Ionis/AstraZeneca	2023	TTR
Olezarsen	Tryngolza	Ionis Pharmaceuticals	2024	APOC3
Donidalorsen	Dawnzera	Ionis Pharmaceuticals	2025	KLKB1

B

Target	ID	chr	start	end	ASO sequence	RNase H score	GC content	ASO MFE	4 mismatch genes	3 mismatch genes	2 mismatch genes
SOD1	Tofersen	NC_000021.9	31668629	31668649	CAGGATACATTTCTACAGCT	-0.316312356	0.4	-0.8	0	0	0
APOB100	Mipomersen	NC_000002.12	21016630	21016650	GCCTCAGTCTGCTTCGCACC	-0.438978448	0.65	-0.3	0	0	0
KLKB1	Donidalorsen	NC_000004.12	186251565	186251585	TGCAAGTCTCTTGGCAAACA	-0.345786073	0.45	-2	0	0	1
TTR	Inotersen	NC_000018.10	31598713	31598733	CTCTTGGTTACATGAAATCC	0.403416403	0.4	-1.1	1	0	0
APOC3	ASO2694	NC_000011.10	116833045	116833065	AGCTTCTTGTCCAGCTTTAT	-0.583327284	0.4	-1.8	0	0	0
GFAP	zilganersen	NC_000017.11	44909448	44909468	CAGTATTACCTCTACTAGTC	-0.164973392	0.4	-0.2	0	0	0
FUS	Ulefnersen	NC_000016.10	31186181	31186201	GCAATGTCACCTTTCATACC	0.224329296	0.45	0	0	0	0
DGAT2	ION224	NC_000011.10	75785888	75785908	TGCCATTTAATGAGCTTCAC	-0.014613995	0.4	-1.4	0	0	0
PCSK9	AZD8233	NC_000001.11	55042353	55042369	TAATCTCATGTCAGTT	-0.615086151	0.3125	0	1	0	0

Target	ID	1 mismatch genes	0 mismatch genes	mismatch genes name	ASO position	Crab-eating macaque	Mouse	Rat	Pig	Rabbit	Guinea pig
SOD1	Tofersen	1	0	PRDM1	intron	0.95	0.65	0.7	0.6	0.8	0.6
APOB100	Mipomersen	0	0	N/A	intron	0.90	0.75	0.70	0.65	0.90	0.65
KLKB1	Donidalorsen	1	0	CDH4/GABRG3	exon	0.95	0.9	0.85	0.95	0.85	0.95
TTR	Inotersen	0	0	LOC124904277	intron	1	0.65	0.6	0.8	0.8	0.8
APOC3	ASO2694	3	0	CACNA1C/CACNA1C/NEURL1	intron	1.00	0.95	0.95	0.85	0.90	
GFAP	zilganersen	0	0	N/A	intron	0.95	0.7	0.65	0.65	0.65	0.65
FUS	Ulefnersen	2	0	BTRC/ERP44	intron	1	1	1	1	1	1
DGAT2	ION224	3	0	.OC124900690/CAND1.11/RBP.	intron	1	0.75	0.7	0.75	0.8	0.75
PCSK9	AZD8233	0	0	LATS1	intron	1	0.75	0.75	0.69	0.62	

Figure S2. Parameter information of FDA approved ASOs and those in clinical trials within the ClinASO.

(A) FDA approved gapmer ASOs.

(B) Parameter information of ASOs in clinical trials within ClinASO.

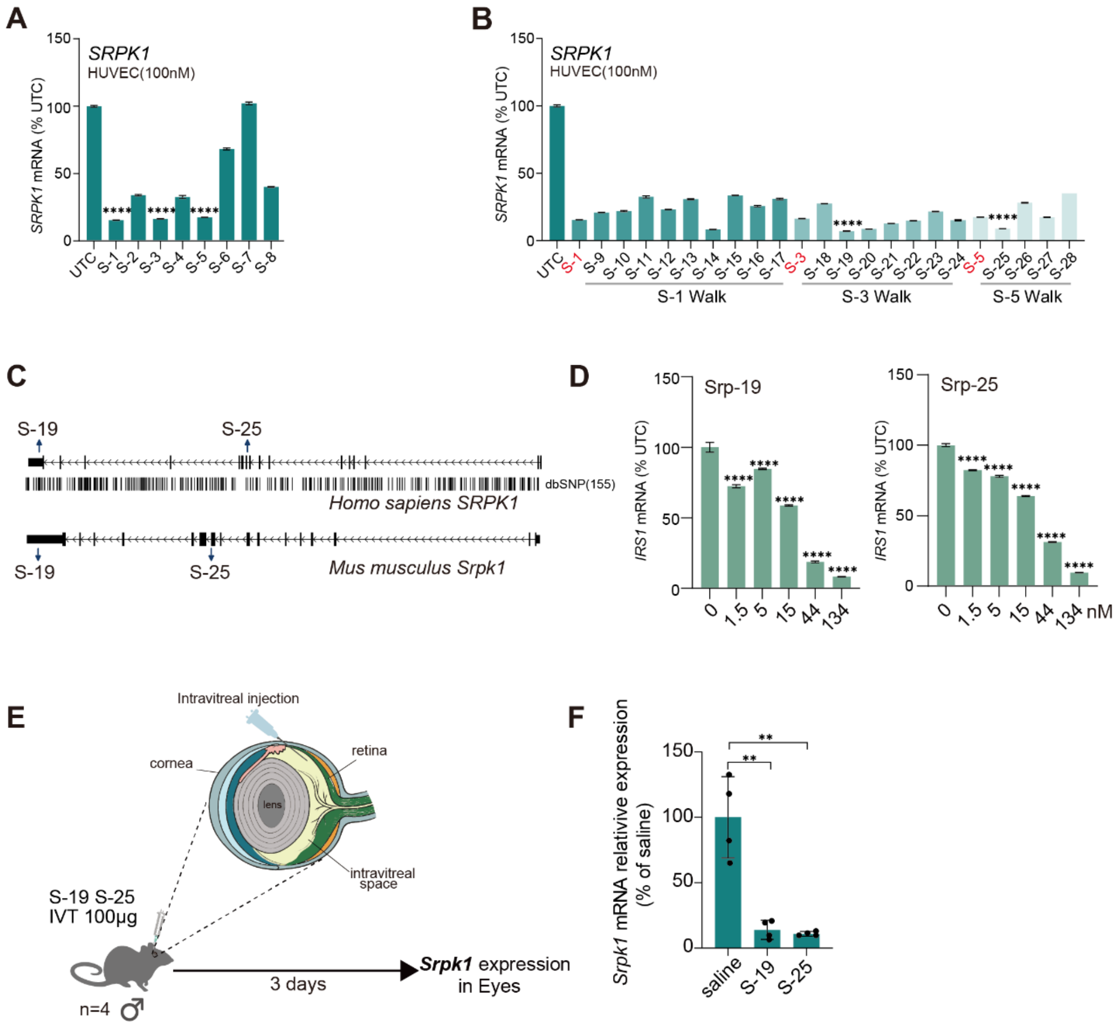

Figure S3. Efficacy of ASOs targeting *SRPK1*.

(A & B) Barplots showing preliminary screening and hotspot identification of ASOs targeting *SRPK1* in HUVECs transfected at 100nM for 24h, measured by RT-qPCR with *β-Actin* as internal control.

(C) Schematic representation of the targeting sites of candidate ASOs (S-19 and S-25) on the homologous *SRPK1* sequence.

(D) Barplots showing quantification of *SRPK1* mRNA knockdown by RT-qPCR with *β-Actin* as internal control in HUVECs 24 h after transfection with increasing concentrations of ASOs. Data are mean ± SD; $*p < 0.05$, $**p < 0.01$, $***p < 0.001$, $****p < 0.0001$ versus control (one-way ANOVA).

(E) Schematic representation of intravitreal injection in mice for evaluating *SRPK1*-targeting ASOs.

(F) Barplots showing *Srpk1* mRNA levels in mouse ocular tissues three days after intravitreal administration of ASOs S-19 or S-25 (100µg per eye), quantified by RT-qPCR with *β-Actin* as internal control. Data are mean ± SD (n = 4 biological replicates)**.** $p < 0.01$ versus saline-treated controls (one-way ANOVA).

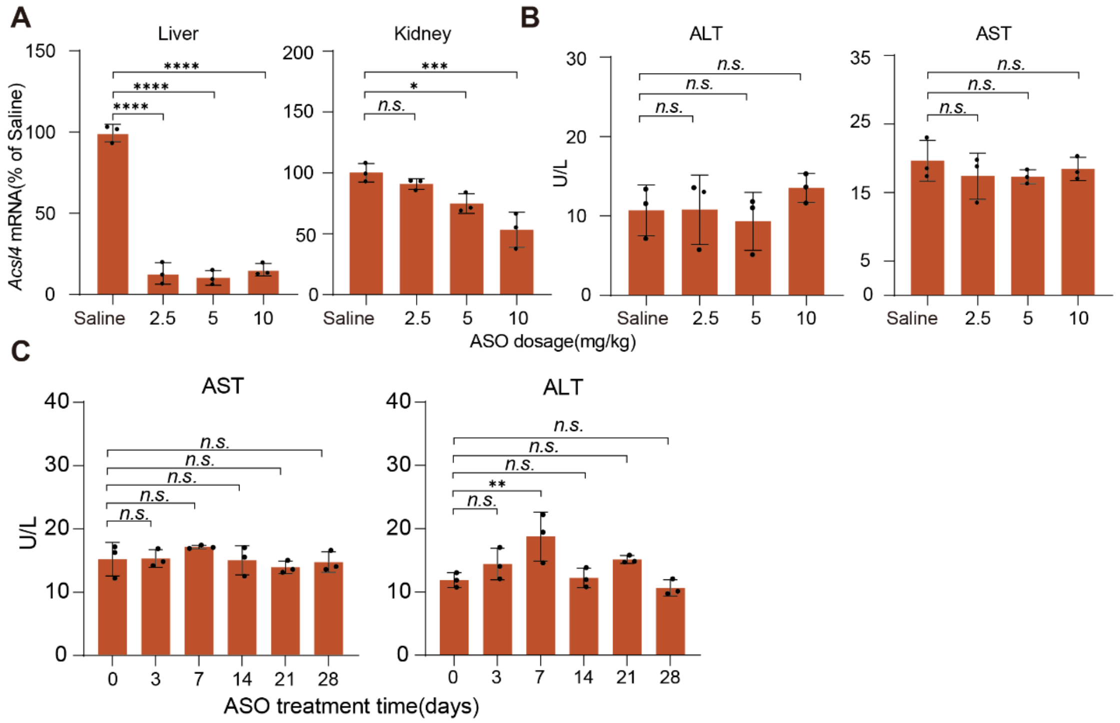

Figure S4. Evaluation of the knockdown efficiency and safety of GA-28c

(A) Barplots showing *ACSL4* mRNA levels in liver and kidney tissues from the experiment shown in (C), quantified by RT-qPCR with *GAPDH* as internal control. Data are presented as mean ± SD; n = 3 biological replicates.

(B) Barplots showing Serum alanine aminotransferase (ALT) and aspartate aminotransferase (AST) levels in mice from the experiment shown in (C). Data are presented as mean ± SD; n = 3 biological replicates.

(C) Barplots showing Serum ALT and AST levels measured at the indicated time points during the treatment of GA-28c. Data are presented as mean ± SD; n = 3 biological replicates.

For panel A, B and C, Statistical significance was determined by one-way ANOVA: *n.s.*: no significance, **p* < 0.05, ***p* < 0.01, ****p* < 0.001, *****p* < 0.0001.

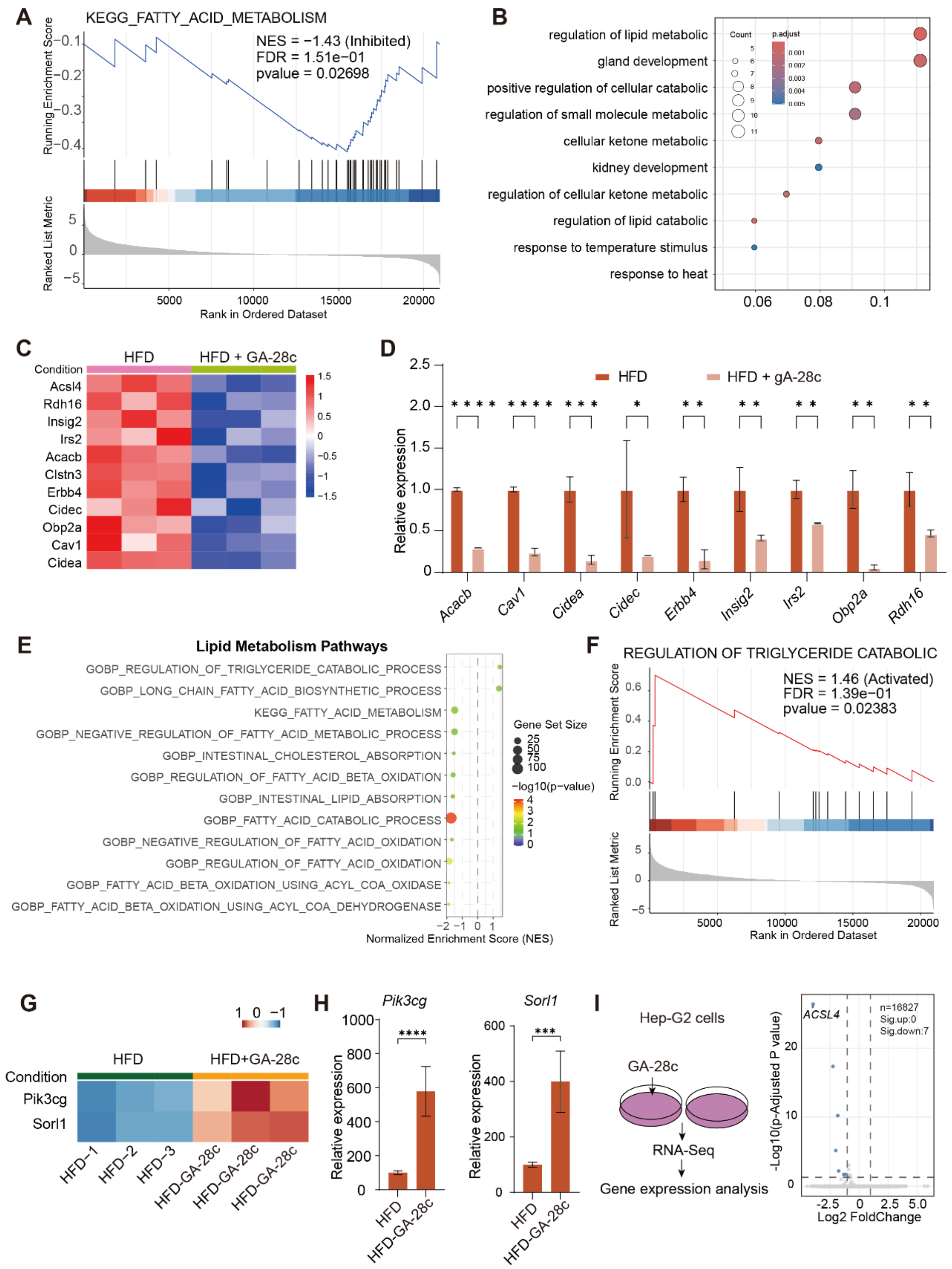

Figure S5. Mechanistic analyses of therapeutic effects of GA-28c in HFD mice

(A) Gene-set enrichment analysis (GSEA) of RNA-seq data (n = 3 biological replicates per group) on KEGG Fatty acid metabolism pathway in HFD and GA-28c-treated mouse livers vs HFD control (NES = –1.43).

(B) Gene Ontology (GO) enrichment map of the down-regulated genes GA-28c-treated mouse livers vs HFD control.

(C) Heatmap showing gene expression profiles involved in lipid-metabolism of 3 individual mice.

(D) Barplots showing RT-qPCR with *GAPDH* as internal control validation of expression 9 representative genes from panel C. Data are presented as mean ± SD; n = 3. Student's *t* test, * $p < 0.05$, ** $p < 0.01$, *** $p < 0.001$, **** $p < 0.0001$.

(E) Bubble plot summarizing GSEA of all lipid-related KEGG pathways for RNA-seq of HFD and GA-28c-treated vs HFD mice.

(F) GSEA analysis for the GO term Regulation of triglyceride catabolic process for livers of HFD and GA-28c-treated vs HFD mice.

(G) Heat-map of the *Pik3cg* and *Sorl1* expression from (F).

(H) Barplots showing RT-qPCR results of *Pik3cg* and *Sorl1* induction with *GAPDH* as internal control. Bars represent fold-change. Data are presented as mean ± SD; n = 3. Student's *t* test, ***$p < 0.001$, ****$p < 0.0001$.

(I) Cartoon showing Off-target effect of GA-28c (44nM) in HepG2 cells and volcano plot of RNA-seq. (n = 3, |log2FC| > 1 and *p* adjust value < 0.05)).

Table S1: Information on target-specific antisense oligonucleotides against *ACSL4, SRPK1, IRS1,* and *PCSK9*. Due to its length, this supplemental table cannot fit within two 8.5" × 11" pages and has therefore been uploaded as a separate Excel file.

Table S2：Sequences of antisense oligonucleotides designed for ACSL4 and SRPK1(Walk)

Gene	ACSL4	SRPK1	
A-19 Walk		S-1 Walk	
ID	Sequence	ID	Sequence
A-22	GCGTTTTTCTTCTTGGCATT	S-9	TGCCACAATGAGCTTGCGAG
A-23	AGCGTTTTTCTTCTTGGCAT	S-10	GCCACAATGAGCTTGCGAGG
A-24	TAGCGTTTTTCTTCTTGGCA	S-11	CCACAATGAGCTTGCGAGGC
A-25	ATAGCGTTTTTCTTCTTGGC	S-12	CACAATGAGCTTGCGAGGCA
A-26	CATAGCGTTTTTCTTCTTGG	S-13	CAATGAGCTTGCGAGGCACC
A-19	CCATAGCGTTTTTCTTCTTG	S-14	AATGAGCTTGCGAGGCACCT
A-27	GCCATAGCGTTTTTCTTCTT	S-15	ATGAGCTTGCGAGGCACCTT
A-28	TGCCATAGCGTTTTTCTTCT	S-16	TGAGCTTGCGAGGCACCTTC
A-29	TTGCCATAGCGTTTTTCTTC	S-17	GAGCTTGCGAGGCACCTTCC
A-30	TTTGCCATAGCGTTTTTCTT	S-3 Walk	
A-31	CTTTGCCATAGCGTTTTTCT	ID	Sequence
A-5 Walk		S-18	CCGCGGGATTCAGCGCCGAC
ID	Sequence	S-19	TCCGCGGGATTCAGCGCCGA
A-32	TGGCCATGTTTAAGATTCTT	S-20	GTCCGCGGGATTCAGCGCCG
A-33	GGCCATGTTTAAGATTCTTC	S-21	CGTCCGCGGGATTCAGCGCC
A-5	GCCATGTTTAAGATTCTTCT	S-22	TCGTCCGCGGGATTCAGCGC
A-34	CCATGTTTAAGATTCTTCTA	S-23	GTCGTCCGCGGGATTCAGCG
A-35	CATGTTTAAGATTCTTCTAA	S-24	GGGTCGTCCGCGGGATTCAG
A-36	ATGTTTAAGATTCTTCTAAT	S-5 Walk	
A-37	TGTTTAAGATTCTTCTAATG	ID	Sequence
A-38	GTTTAAGATTCTTCTAATGC	S-25	CTGCATTCGCTTCTCTAGTA
A-39	TTTAAGATTCTTCTAATGCA	S-26	GCATTCGCTTCTCTAGTAAT
		S-27	CATTCGCTTCTCTAGTAATT
		S-28	ATTCGCTTCTCTAGTAATTC

Table S3: ***PCSK9 ACSL4* Low-Efficiency Score ASOs.** Due to its length, this supplemental table cannot fit within two 8.5" × 11" pages and has therefore been uploaded as a separate Excel file.
