# Supplementary material for: ClinASO: An open-source platform for rapid drug discovery of gapmer antisense oligonucleotides
Source: Mol Ther Nucleic Acids. 2026 Apr 16;37(2):102933. doi: 10.1016/j.omtn.2026.102933 (PMC13156736; doi:10.1016/j.omtn.2026.102933)
Supplement: Document S2. Article plus supplemental information [file mmc4.pdf]

Molecular Therapy
Nucleic Acids
Original Article

# ClinASO: An open-source platform for rapid drug discovery of gapmer antisense oligonucleotides

Shunkai Chen,[1,11] Hao Liu,[1,11] Dezi Cong,[2,11] Ao Dong,[1] Yuhang Wang,[2] Jingyi Bi,[1] Shijie Guo,[1] Juan Yang,[3] Xiaolei Wang,[2,4] Guiping Ren,[1] Ke Zhang,[5,6,7] Haisheng Wang,[2,8,9] Fan Lai,[1,2,10] and Yunkun Dang[1,2,10]

[1]MOE Key Laboratory for Southwest Microbial Diversity, Yunnan Key Laboratory of Cell Metabolism and Diseases, Center for Life Science, School of Life Sciences, Yunnan University, Kunming, China; [2]SicaGene Bioscience Co., Ltd., 4th Floor, Building 6, Courtyard 20, Ping'an Road, Mentougou District, Beijing, China; [3]Department of Anesthesiology, First Affiliated Hospital of Kunming Medical University, Kunming, Yunnan Province 650032, China; [4]State Key Laboratory of Applied Organic Chemistry, College of Chemistry and Chemical Engineering, Lanzhou University, 222 S Tianshui Rd, Lanzhou 730000, China; [5]Institute of Neurological and Psychiatric Disorders, Shenzhen Bay Laboratory, Shenzhen, Guangdong 518107, China; [6]Shenzhen Medical Academy of Research and Translation Ph.D. Program, Shenzhen, Guangdong 518107, China; [7]Rare Disease Center, Shenzhen Medical Academy of Research and Translation, Shenzhen, Guangdong 518107, China; [8]Key Laboratory of Organic Chemistry, Institute of Organic Chemistry, Jiangxi Science & Technology Normal University, Nanchang 330013, China; [9]Wenzhou Institute, University of Chinese Academy of Sciences, Wenzhou, Zhejiang 325001, China; [10]Southwest United Graduate School, Kunming, China

**Gapmer antisense oligonucleotides (ASOs) enable sequence-specific degradation of target mRNAs, offering therapeutic access to previously "undruggable" genes and holding great promise for treating chronic and genetic diseases. However, the rapid development of ASO therapeutics remains limited by challenges in rational sequence design and translational validation. Here, we present ClinASO, an integrated computational-experimental platform that unifies key determinants of ASO efficacy—including RNase H1 cleavage preference, SNP avoidance, off-target filtering, and cross-species conservation—into a single, data-driven workflow. This system enables rapid identification of potent ASO leads and direct *in vivo* validation in wild-type animal disease models. Using ClinASO, we efficiently identified potent ASOs against *PCSK9* and *IRS1*, both exhibiting superior silencing activity compared to their clinical counterparts. Furthermore, ClinASO generated a potent ASO targeting *ACSL4* genes implicated in metabolic dysfunction-associated liver disease (MASLD). In multiple human cells, the ASO achieved robust silencing effect. Notably, by conjugating GalNAc, this ASO demonstrated durable, liver-specific knockdown, significantly ameliorating hepatic steatosis and normalizing systemic lipid profiles in MASLD mouse model. Together, these findings establish ClinASO as an efficient, experimentally validated online tool for the rational design and rapid development of translatable ASO therapeutics.**

## INTRODUCTION

Antisense oligonucleotides (ASOs) are short, single-stranded nucleic acid molecules (15–30 nucleotides) that bind complementary RNA sequences via Watson-Crick base pairing to modulate gene expression at the RNA level.[1] Depending on their chemical structure and design, ASOs can induce mRNA degradation, alter pre-mRNA splicing, or inhibit translation, thereby offering a versatile and programmable means of gene regulation.[2–4] In addition, their single-stranded nature and lower molecular weight enable efficient cellular uptake through passive diffusion or by forming transient complexes with serum proteins such as albumin, thereby reducing dependence on complex delivery systems.[5,6] Over the past decade, ASOs have emerged as a powerful therapeutic modality for metabolic, ophthalmic, and neurodegenerative diseases.[7–11]

Among various ASO modalities, gapmer ASOs represent the most widely adopted design for gene silencing, with 7 drugs approved for clinical use and over a hundred in clinical development.[12,13] Structurally, they consist of a central DNA "gap" flanked by chemically modified ribonucleotide "wings".[14,15] Upon hybridization with target RNA in the nucleus, the DNA/RNA heteroduplex recruits endogenous RNase H1, which cleaves the RNA strand and thus triggers RNA degradation.[16] Therapeutic ASOs contain a series of chemical modifications to improve the stability, efficacy, and safety. Phosphorothioate (PS) backbone linkages, in which a non-bridging oxygen atom is replaced by sulfur, enhance resistance to nuclease

Received 15 December 2025; accepted 14 April 2026;
https://doi.org/10.1016/j.omtn.2026.102933.

[11]These authors contributed equally

**Correspondence:** Haisheng Wang, SicaGene Bioscience Co., Ltd. 4th Floor, Building 6, Courtyard 20, Ping'an Road, Mentougou District, Beijing, China.
**E-mail:** haishengwang@sicagene.com
**Correspondence:** Fan Lai, MOE Key Laboratory for Southwest Microbial Diversity, Yunnan Key Laboratory of Cell Metabolism and Diseases, Center for Life Science, School of Life Sciences, Yunnan University, Kunming, China.
**E-mail:** fanlai@ynu.edu.cn
**Correspondence:** Yunkun Dang, MOE Key Laboratory for Southwest Microbial Diversity, Yunnan Key Laboratory of Cell Metabolism and Diseases, Center for Life Science, School of Life Sciences, Yunnan University, Kunming, China.
**E-mail:** ykdang@ynu.edu.cn


© 2026 The Authors.
Published by Elsevier Inc. on behalf of The American Society of Gene and Cell Therapy.
This is an open access article under the CC BY license (http://creativecommons.org/licenses/by/4.0/).

and promote plasma protein binding, thereby extending circulation half-life and improving tissue distribution.[17,18] Additionally, 2′-sugar modifications—such as 2′-O-methyl (2′-OMe), 2′-O-methoxyethyl (2′-MOE), and constrained ethyl (2′-cEt)—introduced in the ASO "wing" regions greatly improve RNA-binding affinity, duplex stability, and reduce immunogenicity.[15,19–22] For liver-directed therapeutics, conjugation with *N*-acetylgalactosamine (GalNAc) confer specific binding with the asialoglycoprotein receptor (*ASGPR*) that is highly expressed on hepatocytes, thus ensuring efficient and selective uptake.[23–25] Together, these advances enable gapmer ASO drugs to achieve long-acting gene silencing, often with dosing intervals extending to several weeks or even months.

Despite their therapeutic promise, preclinical development of gapmer ASOs remains complex and time consuming largely due to ASO sequence selection. Although gapmer ASOs can be designed to, in principle, target any region of a pre-mRNA,[26] multiple factors can influence silencing efficiency, including hybridization thermodynamics,[27] RNA accessibility,[28] off-target,[29] and ASO self-complementarity.[30] Moreover, RNase H1 exhibits intrinsic sequence preferences that affect cleavage efficiency, further complicating target site identification.[31] In addition, the presence of single-nucleotide polymorphisms (SNPs) within the target RNA sequence can substantially impact ASO efficacy. Even a single mismatch can lead to reduced efficacy in specific patient populations.[32–34] Finally, preclinical validation often relies on humanized mouse models expressing cDNA constructs that lack introns and regulatory elements. Because gapmer ASOs primarily act on pre-mRNA in the nucleus,[26] these cDNA-based models fail to accurately recapitulate endogenous splicing and transcriptional dynamics, potentially leading to misleading efficacy evaluations and poor translational predictability.[35]

Although gapmer ASO has been established as potent therapeutics, existing design tools remain limited and not undergone systematic validation across *in vitro* and *in vivo* models,[36–38] thereby impeding their use in ASO drug discovery. Given the highly conserved transcriptional machinery among mammals where homologous genes often share similar exon-intron architecture, we developed ClinASO, an integrated computational platform designed to accelerate the discovery and evaluation of clinically translatable gapmer ASOs. ClinASO employs a unified scoring algorithm that integrates multiple determinants of gapmer ASO efficacy and safety, including SNP avoidance, ASO secondary structure stability, RNase H1 cleavage preference, off-target prediction, and cross-species sequence conservation for *in vivo* validation. This comprehensive design strategy enables rapid identification of potent ASOs for human therapeutic targets while ensuring translational validation with wide-type animal models, thereby bridging the gap between *in silico* design and *in vivo* validation.

## RESULTS

### ClinASO: An integrated online tool for the rational design of gapmer ASO sequences

To systematically enhance design efficiency and improve the success rate of clinical gapmer ASO development, we created ClinASO (www.gapmerasodesign.com), an integrated web-based platform that incorporates all key parameters for rational ASO sequence selection.

For a given target gene, the platform first identifies and excludes sites containing annotated SNPs using the NCBI dbSNP(build 155) that curates ∼1.0 billion SNPs, ensuring population-wide coverage.[39] The remaining SNP-free sequence sections are segmented using a 20-nucleotide sliding window (corresponding to a canonical 5-10-5 gapmer ASO structure with 2′-MOE modifications at wing regions). Each candidate sequence is then evaluated for intrinsic secondary structure stability and potential off-target binding (defined as < 4 mismatches by BLAST).[40] Candidate sequences passing these filters are subsequently analyzed using an RNase H1 cleavage preference module, which prioritizes motifs associated with high catalytic activity and exhibits as RNase H1 score (lower the value, higher the putative cleavage efficiency).[31] Finally, ClinASO performs homology comparisons between human target sequences and orthologous genes (with value 1 as 100% sequence identity) in commonly used preclinical species—including monkey (*Macaca fascicularis*), mouse (*Mus musculus*), rat (*Rattus norvegicus*), rabbit (*Oryctolagus cuniculus*), pig (*Sus scrofa*), and guinea pig (*Cavia porcellus*)—to ensure translational compatibility for *in vivo* testing (Figure 1A). In addition to automated ASO sequence design, the platform also provides standalone analytical tools, including homology mapping, SNP-specific sequence query, and off-target prediction, thus facilitating the evaluation of ASO candidates from users (Figures 1B and S1).

To validate the performance of the ClinASO platform, we applied it to design gapmer ASOs targeting 4 disease-associated genes—*PCSK9*, *ACSL4*, *IRS1*, and *SRPK1*—which have been previously implicated as therapeutic targets in liver and ophthalmic disorders (Figure 1C). ClinASO generates comprehensive output reports summarizing all critical sequence parameters, including genomic position, ASO sequence, predicted cleavage efficiency (RNase H1 score; lower values indicate higher predicted activity), GC content, secondary structure stability (minimum free energy or MFE, higher values indicate lower structural potentials), potential off-target matches on annotated transcripts, and sequence homology across multiple preclinical species. An example output (Figure 1D) illustrates the detailed tabular results, where each candidate ASO is ranked according to its composite score. This systematic presentation enables users to efficiently prioritize optimal sequences for experimental validation. To preliminarily evaluate the effectiveness of ClinASO, we analyzed the sequence features of 9 clinically approved ASO drugs. Most of these ASOs exhibit GC content between 0.3 and 0.6, MFE > −2, RNase H1 score <0.2, and very few potential off-targets, suggesting that these parameters are important determinants for selecting potent ASO sequences (Figure S2).

To accelerate ASO translation, we proposed an end-to-end workflow comprising two major stages: *in vitro* screening in human cells and *in vivo* validation in animal models, subdivided into 5 key steps (Figure 1E). In the *in vitro* stage, ClinASO-designed 5-10-5 gapmer

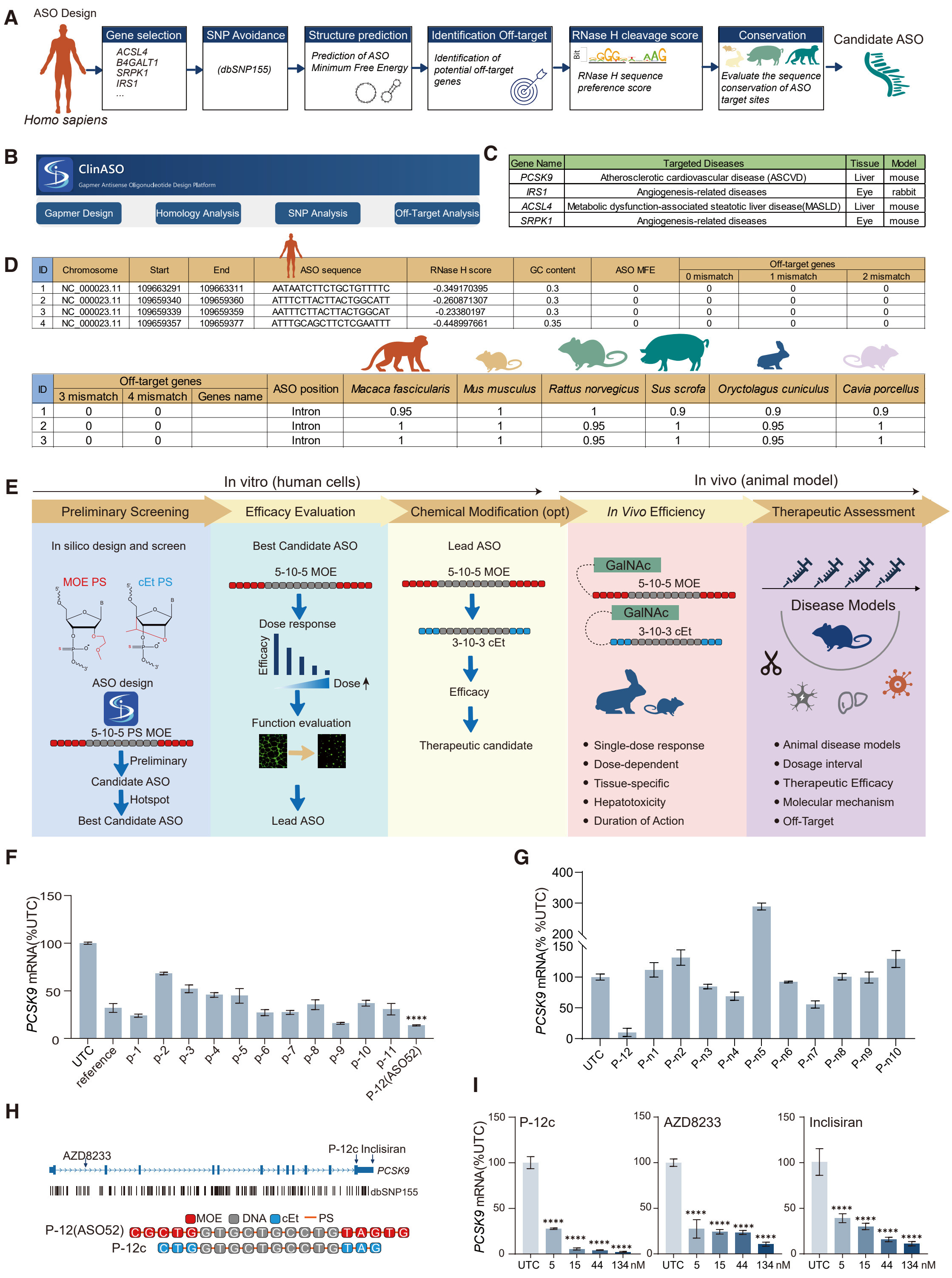


**Figure 1. Overview of the ClinASO workflow and the screening process for ASO**

(A) Schematic workflow of the core design parameters integrated into the ClinASO platform. SNP data from dbSNP build 155; ASO secondary structure analyses using RNAfold (2.7.0)[41]; off-target prediction using Blastn[42]; RNase H1 sequence preference (RNase H1 score) derived from published data.[31] (B) The interface of ClinASO. (C) Table showing the four target genes (*PCSK9*, *IRS1*, *ACSL4*, and *SRPK1*) for platform validation, with related diseases and animal models. (D) Representative output table of

*(legend continued on next page)*

ASOs (full PS linkages with 2′-MOE-modified wings) are synthesized and screened in 3 steps. Step 1: preliminary single-dose assays rapidly identify active sequences, followed by hotspot sequence-walking to optimize local regions and define the lead ASO. Step 2: dose-response and functional assays quantify silencing potency and biological effects. Step 3 (optional): chemical refinement introduces 2′-cEt modifications or GalNAc conjugation to enhance stability and tissue targeting. In the *in vivo* stage, optimized ASOs are tested in sequence-conserved animal models using appropriate delivery routes. Step 4: single-dose studies assess tissue-specific knockdown and pharmacokinetics. Step 5: top performers proceed to efficacy and safety evaluation.

### ClinASO enables efficient identification of potent ASO against *PCSK9*

*PCSK9* (proprotein convertase subtilisin/kexin type 9) is a pivotal regulator of cholesterol homeostasis. By binding to low-density lipoprotein receptors (LDLRs) on hepatocytes and directing them toward lysosomal degradation, *PCSK9* reduces LDLR surface availability, thereby elevating circulating LDL cholesterol (LDL-C) levels.[43–45] Gain-of-function mutations in *PCSK9* result in familial hypercholesterolemia (FH), whereas loss-of-function variants are associated with lifelong reductions in LDL-C and an approximately 50%–80% decrease in cardiovascular disease (CVD) risk.[44,46] Owing to this strong human genetic validation, *PCSK9* has been established as a premier therapeutic target for lipid-lowering interventions. To date, two oligonucleotide-based therapeutics have been developed against *PCSK9*: Inclisiran, a small interfering RNA (siRNA) therapy approved by the FDA, and AZD8233, an ASO currently completing phase 2 clinical trials.[47–50]

To assess the gene-silencing efficacy of ASOs designed using the ClinASO platform, 12 sequence candidates targeting *PCSK9* were selected and synthesized as 5-10-5 gapmer ASOs with full PS linkages and 2′-MOE modifications at the wings. As a reference, the AZD8233 sequence was included, extended to 20 nucleotides by adding two bases at each end, while substituting its original 2′-cEt modification with 2′-MOE. Each ASO was transfected individually into HepG2 cells at 100 nM. Among the candidates, P-2, P-9, and P-12 exhibited superior silencing activity compared to the control (Figure 1F). In contrast, sequences with unfavorable parameters (MFE $< -5$, GC% 0.2–0.3 or 0.6–0.75, >10 putative off-target genes with 4-base mismatches, unpreferred RNase H1 score) predicted by ClinASO (P-n1 to P-n10) displayed markedly low silencing activity (Figure 1G). The lead sequence P-12, later designated ASO52 by conjugating GalNAc for liver-specific delivery, demonstrated potent and durable therapeutic efficacy in an obese mouse model.[51] Remarkably, ASO52 achieved lipid-lowering effects equivalent to Inclisiran and maintained its activity for over 5 weeks following a single 20 mg/kg dose, with no observable hepatotoxicity, indicating both robust efficacy and favorable safety.[51]

To strictly evaluate the silencing effects conferred by the sequence between P-12 (ASO52) and AZD8233, which is in a 3-10-3 design pattern with 2′-cEt modification at each wing, we introduced the same modification into the P-12 sequence and removed 2 bases at each end (Figure 1H), generating P-12c. In human HepG2 cells, P-12c displayed comparable silencing efficacy to AZD8233 and Inclisiran at a 5 nM concentration, while exhibiting markedly superior efficiency at higher doses (15 nM, 44 nM, and 134 nM) (Figure 1I). Collectively, these results suggest that ClinASO can efficiently identify potent gapmer ASO sequences targeting *PCSK9*, enabling the generation of clinically competitive leads through rational, data-driven design.

### ClinASO enables efficient identification of potent ASO against *IRS1*

To evaluate the broader applicability of ClinASO for disease-relevant targets, we next focused on insulin receptor substrate 1 (*IRS1*), a key adaptor protein mediating insulin and IGF-1 signaling.[52,53] Dysregulation of *IRS1* has been increasingly implicated in retinal vascular pathologies, especially in metabolic conditions such as diabetic retinopathy.[54] Notably, Aganirsen (GS-101), a 25-mer phosphorothioate antisense oligonucleotide designed to inhibit *IRS1* expression, has demonstrated significant anti-angiogenic activity in preclinical and clinical studies.[55–57] Aganirsen is among the few ophthalmic ASOs to reach phase 2/3 clinical trials, where it was shown to reduce corneal and retinal neovascularization in multiple animal models, inhibit endothelial cell proliferation and migration, and prevent pathological angiogenesis triggered by inflammation or hypoxia.[57] This makes *IRS1* an ideal benchmark target for validating ClinASO's ability to design therapeutically relevant ophthalmic gapmer ASOs.[58]

For *IRS1*, 17 sequence candidates generated by ClinASO were synthesized in the 5-10-5 MOE pattern and screened in human umbilical vein endothelial cells (HUVECs) at 100 nM, which revealed 6 effective sequence candidates with superior activity to Aganirsen, including I-5 (55.45%), I-8 (60.96%), I-9 (67.24%), I-10 (57.14%), I-15 (60.07%), and I-17 (46.39%) (all $p < 0.0001$) (Figures 2A and 2B). Given the established role of *IRS1* in endothelial angiogenesis,[59] we employed the HUVEC tube formation assay as a functional readout to assess angiogenic inhibition upon *IRS1* knockdown (Figure 2C). Six ASO candidates targeting human-rabbit conserved *IRS1* sequences were incubated with HUVECs under free-uptake conditions across a concentration gradient. Compared with

the ClinASO. (E) Workflow for discovery and validation of therapeutic gapmer ASO. (F) Barplots showing preliminary screening of ASOs targeting *PCSK9* in HepG2 cells transfected at 100 nM for 24 h, measured by quantitative reverse transcription PCR(RT-qPCR) with *GAPDH* as internal control. UTC, untreated control. (G) Barplots showing quantification of *PCSK9* mRNA knockdown by ASO with unfavorable parameters predicted by ClinASO. (H) Cartoon depicts the binding site and sequence structures of P-12 (ASO52), P-12c. (I) Barplots showing quantification of *PCSK9* mRNA knockdown by RT-qPCR with *GAPDH* as internal control in HepG2 24 h after transfection with increasing concentrations of ASOs. Data are mean ± SD; *$p < 0.05$, **$p < 0.01$, ***$p < 0.001$, ****$p < 0.0001$ versus UTC (one-way ANOVA).

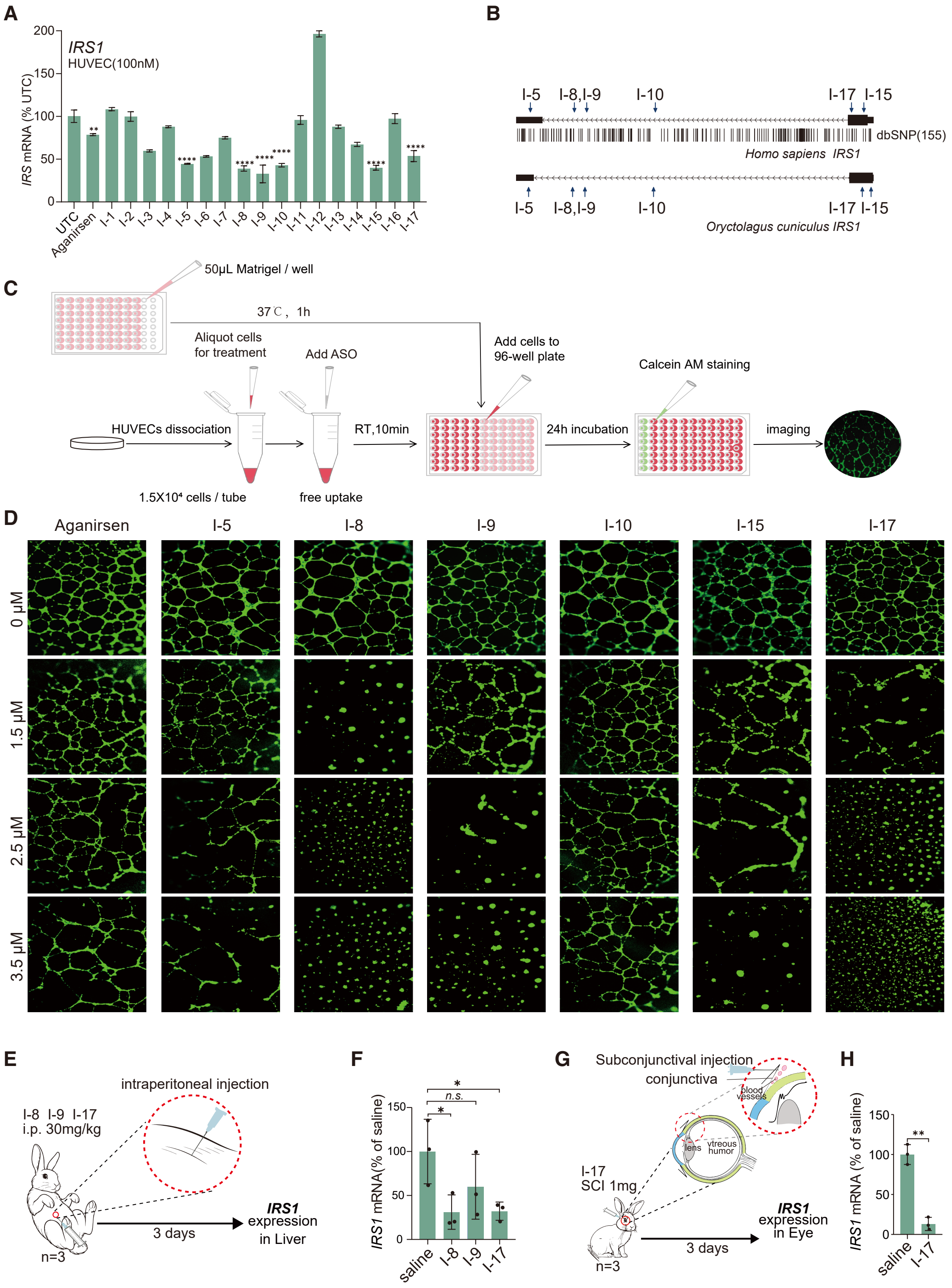


**Figure 2. *in vitro* and *in vivo* efficacy of ASOs targeting *IRS1***

(A) Barplots showing preliminary screening of 17 ASOs targeting *IRS1* in HUVEC cells transfected at 100 nM for 24 h, measured by RT-qPCR with β-actin as internal control. (B) Targeting sites of 6 candidate ASOs within conserved regions of human-rabbit *IRS1* mRNA. (C) Schematic illustration of the HUVEC tube formation assay being used to

*(legend continued on next page)*

untreated controls, I-8, I-9, and I-17 induced significant, dose-dependent suppression of endothelial tube formation (Figure 2D). Together, these results establish a robust foundation for further *in vivo* validation of ASO-based treatments for ophthalmic angiogenic diseases by targeting *IRS1*.

To evaluate the *in vivo* silencing efficacy of ClinASO-designed ASOs targeting *IRS1*, we first tested the 3 human-rabbit homologous ASO candidates in a young rabbit model via systemic intraperitoneal administration (30 mg/kg) (Figure 2E). Hepatic tissues collected 3 days post-injection revealed significant *IRS1* silencing by I-8 and I-17, with knockdown efficiencies of 68.83% and 67.85%, respectively ($p < 0.05$), while I-9 exhibited comparatively weaker activity (Figure 2F). To directly assess ophthalmic efficacy, the most potent candidate, I-17, was delivered via subconjunctival injection into rabbit eyes (Figure 2G). Corneal tissues harvested 3 days post-injection exhibited a substantial reduction in *IRS1* expression (86.8%, $p < 0.0001$) (Figure 2H). Collectively, these results demonstrate that ClinASO-designed ASOs maintain potent, specific, and cross-species gene-silencing activity against *IRS1* both *in vitro* and *in vivo*.

To further assess the design efficiency of ClinASO in ophthalmic diseases targets, we selected *SRPK1* (serine/arginine protein kinase 1), a key regulator of ophthalmic angiogenesis through VEGF signaling and alternative splicing of angiogenesis-related genes,[60,61] for which no ASO therapy currently exists. From 8 ClinASO-designed candidates, 3 exhibited strong silencing activity *in vitro* (Figure S3A). Subsequent base-walk optimization identified two lead ASOs, S-19 and S-25, both showing clear dose-dependent knockdown (Figures S3B–S3D). For *in vivo* validation, S-19 and S-25—targeting human-mouse conserved regions—were administered via intravitreal injection (100 μg per eye) (Figure S3E). Ocular tissues were collected 3 days post-injection for transcript quantification by RT-qPCR. Both ASOs achieved robust silencing of *Srpk1* mRNA, with reductions of 86.00% and 89.12%, respectively ($p < 0.01$) (Figure S3F). Together, these results demonstrate that ClinASO-designed gapmer ASOs can effectively and specifically silence ocular target genes both *in vitro* and in *vivo* in the animal model, supporting the platform's applicability for angiogenesis-related ophthalmic disorders.

### Gapmer ASO against *ACSL4* exhibited potent silencing efficacy *in vitro*

Metabolic dysfunction-associated steatotic liver disease (MASLD) is now the most common chronic liver disorder globally, affecting up to 38% of adults.[62] The condition is marked by hepatic steatosis (lipid droplet accumulation in hepatocytes), leading to metabolic stress and liver injury.[63] Long-chain acyl-CoA synthetase 4 (*ACSL4*) has recently been identified as a key regulator of hepatic lipid metabolism and a promising therapeutic target. By preferentially activating polyunsaturated fatty acids such as arachidonic acid, *ACSL4* influences phospholipid remodeling and lipid signaling. Its upregulation in MASLD promotes a pathogenic feedback loop with TGFβ1/Smad3, suppressing PGC-1α and impairing mitochondrial β-oxidation.[64,65] Conversely, genetic or pharmacologic inhibition of *Acsl4* markedly alleviates hepatic steatosis in mice.[64,65] Thus, *Acsl4* represents an attractive target for durable, sequence-specific silencing using gapmer ASO therapy.

For *ACSL4*, 20 ASO sequences generated by ClinASO targeting human-mouse conserved regions were synthesized in 5-10-5 MOE pattern and transfected into HepG2 cells at 15 nM using the calcium phosphate method. 24 h post-transfection, mRNA levels were quantified relative to a transfection reagent-only control using RT-qPCR. The initial screening identified A-5 (61.96%, $p < 0.0001$) and A-19 (62.97%, $p < 0.0001$) as the most potent sequences (Figure 3A). Subsequent sequence walking around these hotspots yielded optimized variants with enhanced activity (Figure 3B), including A-28 (76.73%, $p < 0.0001$), A-29 (66.57%, $p < 0.0001$), and A-31 (56.00%, $p < 0.0001$). In contrast, sequences with unfavorable parameters (MFE < −5, GC% 0.2–0.3 or 0.6–0.75, >10 putative off-target genes with 4-base mismatches, unpreferred RNase H1 score) predicted by ClinASO (A-n1 to A-n10) displayed markedly reduced silencing activity (Figure 3C).

To ensure the silencing effect of these candidates was not cell-line specific, we systematically assessed 3 lead candidates (A-28, A-29, and A-31) across multiple human and mouse cell lines, including Huh7, HEK293T, and mouse Hepa1-6 cells, in addition to the initial HepG2 screening. At 15 nM, A-28 exhibited the most consistent and potent knockdown across species and cell types—reducing *ACSL4* mRNA levels by 62.99% in Huh7, 60.35% in HEK293T, and 49.79% in HepG2 cells (all $p < 0.0001$), while maintaining comparable efficacy in Hepa1-6 cells (Figure 3E). Collectively these results identify A-28 as a robust and broadly active lead ASO suitable for subsequent chemical optimization.

Compared with 2′-MOE, the 2′-cEt modification represents a next-generation ASO chemistry with higher RNA-binding affinity and improved pharmacokinetics, enabling potent efficacy at lower doses and reducing hepatotoxicity risk.[21] Guided by this rationale, we generated three 16-mer cEt-modified ASOs (3-10-3 structure)—c1,

evaluate angiogenic inhibition following *IRS1* knockdown. (D) Representative images showing dose-dependent suppression of endothelial tube formation by *IRS1*-targeting ASOs under free-uptake conditions. (E) Schematic representation of systemic intraperitoneal injection in young rabbits for initial evaluation of *IRS1*-targeting ASOs. (F) Barplots showing *IRS1* mRNA levels in rabbit hepatic tissues 3 days after intraperitoneal administration of ASOs I-8, I-9, or I-17 (30 mg/kg), determined by RT-qPCR with β-actin as internal control. Data are mean ± SD ($n$ = 3 biological replicates). $p < 0.05$ versus control (one-way ANOVA). (G) Schematic representation of subconjunctival injection in rabbit eyes for local ocular evaluation of the lead *IRS1*-targeting ASO, I-17. (H) Barplots showing *IRS1* mRNA levels in rabbit corneal tissues three days after subconjunctival administration of ASO I-17, measured by RT-qPCR with β-actin as internal control. Data are mean ± SD ($n$ = 3 biological replicates); $p < 0.01$ versus control (unpaired $t$ test). For (A), (F), and (H), Statistical significance was determined by one-way ANOVA: n.s.: no significance, *$p < 0.05$, **$p < 0.01$, ***$p < 0.001$, ****$p < 0.0001$.

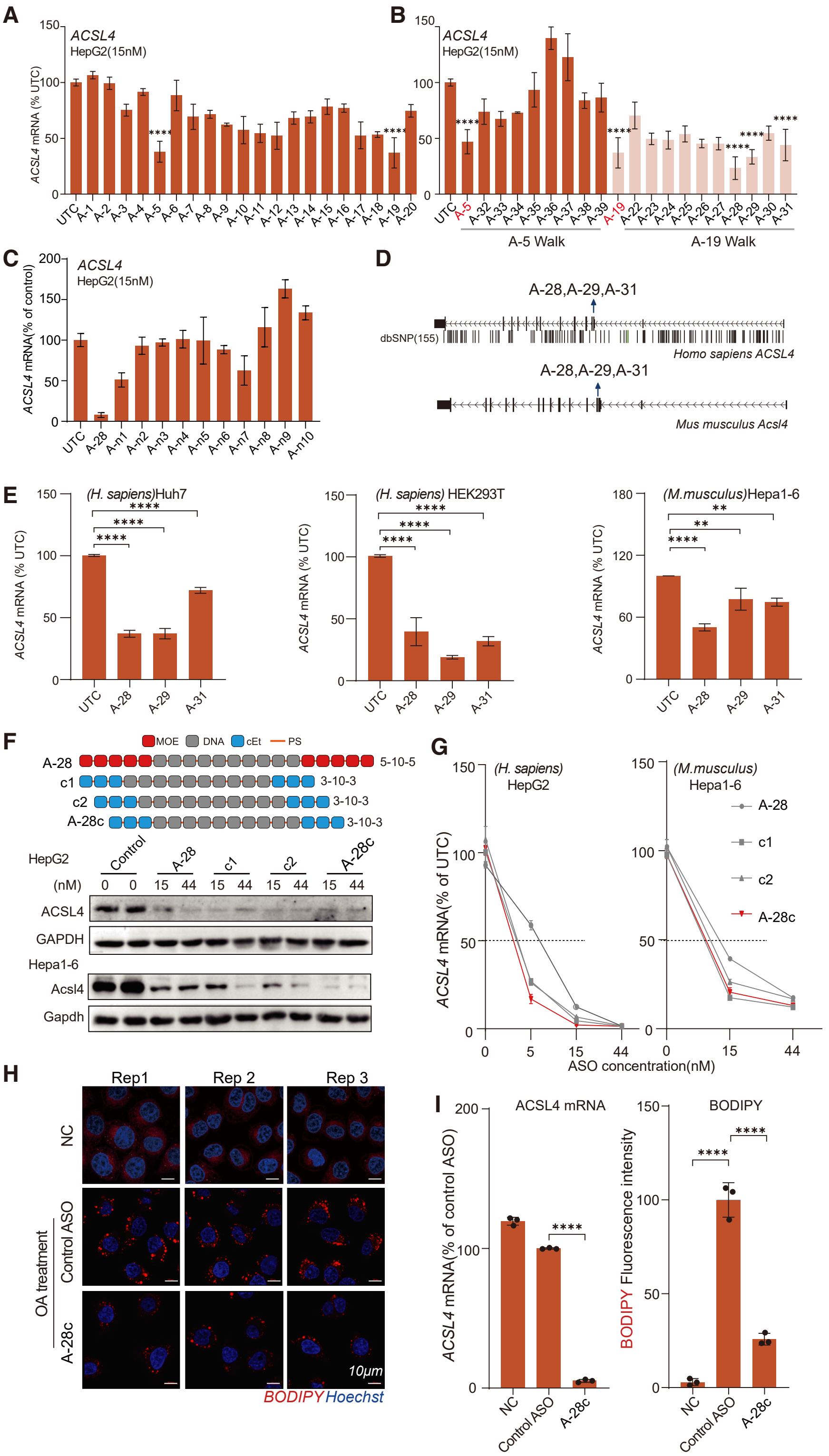


**Figure 3. Dose-response relationship and constrained ethyl optimization of ASO candidates targeting *ACSL4***

(A and B) Barplots showing preliminary screening and hotspot identification of ASOs targeting *ACSL4* in HepG2 cells transfected at 15 nM for 24 h, measured by RT-qPCR with *GAPDH* as internal control. (C) Barplots showing quantification of *ACSL4* mRNA knockdown by ASO with unfavorable parameters predicted by ClinASO. (D) Schematic representation of the targeting sites for lead ASO candidates (A-28, A-29, and A-31) targeting conserved regions of the human and mouse *ACSL4*. (E) Barplots showing knockdown efficacy of ASOs in different cell lines 24 h after transfection and measured by RT-qPCR with *GAPDH* as internal control. Data are mean ± SD (*n* = 3 biological replicates). $p < 0.05$ versus control (one-way ANOVA). (F) Cartoon showing the optimization of 5-10-5 (2′-MOE) to 3-10-3 (cEt) ASO (upper) and knockdown efficiency of the ASOs (15 nM and 44 nM) in human HepG2 and mouse Hepa1-6 cell lines measured by western analyses 24 h after transfection (low). (G) Lineplots showing dose-response curves of *ACSL4* mRNA knockdown by A-28, A-28c, c1, and c2 in HepG2 and Hepa1-6 cells 24 h after transfection and measured by RT-qPCR with *GAPDH* as internal control. Data are presented as mean ± SD; *n* = 3 biological replicates. (H) Representative fluorescent images showing intracellular lipid droplets (green, BODIPY stain) and nuclei (blue, Hoechst stain) in HepG2 cells after 24 h treated with 0.25 mM oleic acid (OA) and a scrambled control ASO or A-28c. Scale bars, 10 μm. (I) Barplots *ACSL4* mRNA knockdown efficiency by RT-qPCR with *GAPDH* as internal control (left) and quantitative analysis of lipid droplet area relative to total cell number (right) for (H). Data are mean ± SD (*n* = 3 biological replicates). In (A), (B), D), and (H), statistical significance was determined by one-way ANOVA: n.s.: no significance, *$p < 0.05$, **$p < 0.01$, ***$p < 0.001$, ****$p < 0.0001$.

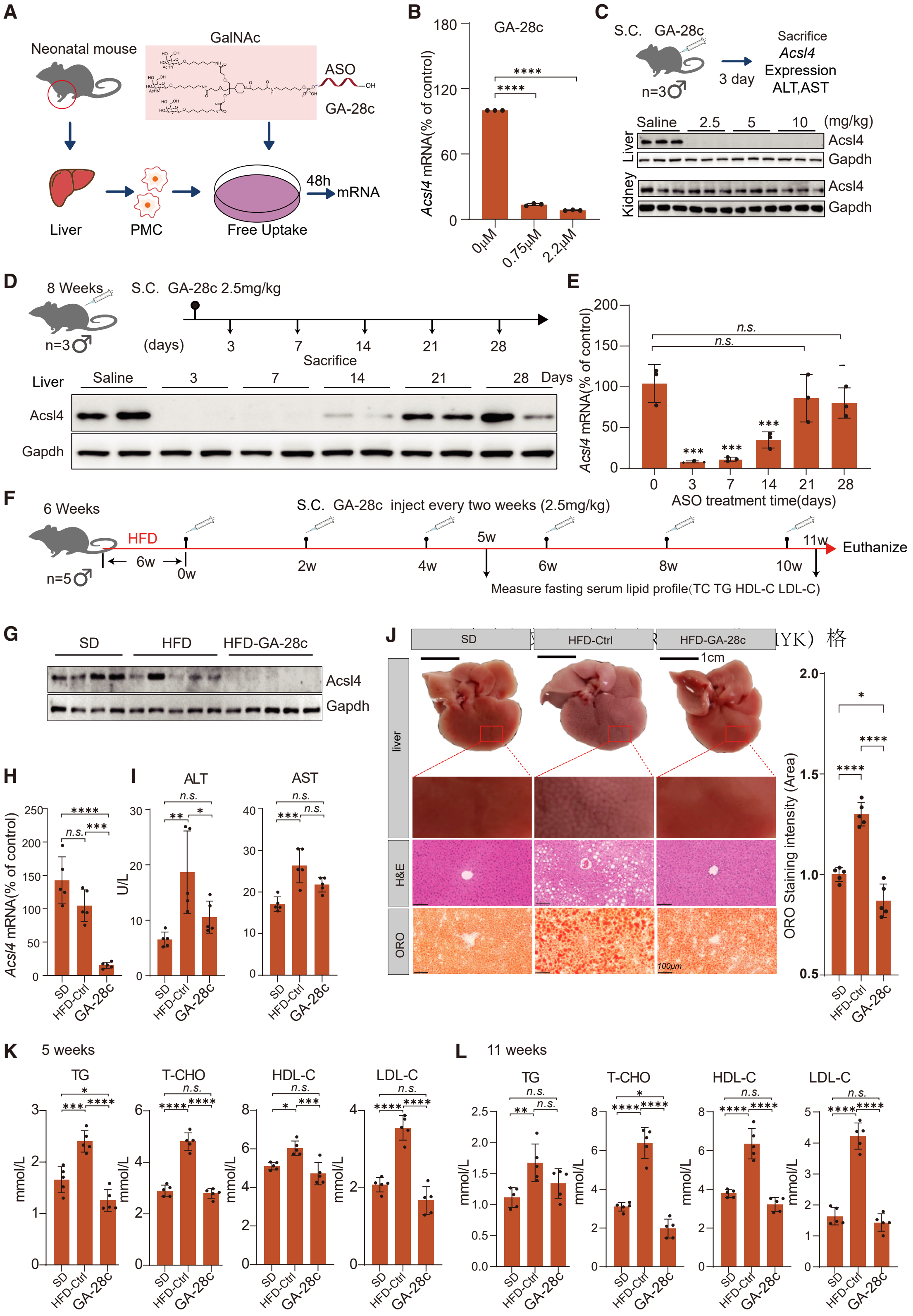


*(legend on next page)*

c2, and A-28c—based on the lead sequence A-28 (Figure 3F). In HepG2 cells, A-28c showed superior, dose-dependent silencing across all concentrations, achieving >97% *ACSL4* mRNA reduction (15 nM: 97.8; 44 nM: 98.6; $p < 0.0001$), confirmed at both mRNA and protein levels (Figures 3F and 3G). Similar results were observed in mouse Hepa1-6 cells, demonstrating cross-species efficacy (15 nM: 79.4; 44 nM: 86.9) (Figures 3F and 3G).

To assess functional outcomes, lipid accumulation was measured in oleic acid (OA)-treated HepG2 cells, a model of hepatic steatosis. OA markedly induced lipid droplets, whereas A-28c treatment significantly reduced their formation relative to scrambled control, as shown by BODIPY staining (Figure 3H). Quantitative analysis confirmed strong *ACSL4* knockdown (94.8%) and reduced lipid accumulation (74.2%; $p < 0.0001$) (Figure 3I). Together, these findings demonstrate that A-28c effectively suppresses *ACSL4* and mitigates lipid deposition, suggesting its potential as a therapeutic candidate for MASLD.

### GalNAc conjugation confers liver-targeting ASOs against *Acsl4*

To assess the preclinical efficacy and safety of ClinASO-designed liver-targeting ASOs, we optimized candidate A-28c by conjugating GalNAc to enhance hepatocyte-specific delivery, generating GA-28c. In primary mouse hepatocytes, GA-28c achieved robust, dose-dependent silencing via free uptake (Figure 4A). GA-28c reduced *Acsl4* expression by 86.40% at 0.75 μM and 91.66% at 2.2 μM ($p < 0.0001$) (Figure 4B). These results confirmed efficient GalNAc-mediated cellular uptake and activity.

*In vivo*, mice received subcutaneous ASO injections, and tissues were analyzed 3 days post-dosing (Figure 4C). GA-28c showed potent and liver-selective silencing, achieving 87.07% ($p < 0.0001$) knockdown at 2.5 mg/kg, confirmed at both mRNA and protein levels (Figures 4C and S4A). Minimal off-target effects in kidneys and stable serum alanine aminotransferase (ALT) and aspartate aminotransferase (AST) levels indicated the safety (Figure S4B). Together these data suggest that GalNAc-conjugated GA-28c achieved efficient liver-specific gene silencing and exhibited excellent safety profiles in *in vivo* models, suggesting their advancement as therapeutic candidates for metabolic and cardiovascular liver diseases.

### GA-28c demonstrates long-acting therapeutic efficacy in a MASLD mouse model

Given the benefits of long-acting feature of ASO drugs to chronic diseases, we next evaluated its duration of action and therapeutic efficacy. Specifically, wild-type C57BL/6 mice received a single subcutaneous injection of GA-28c at various doses, and liver tissues were analyzed on days 3, 7, 14, and 28 (Figure 4D). The ASO induced sustained suppression of *Acsl4* expression, maintaining 65.18% knockdown at day 14 ($p < 0.005$), with protein-level inhibition consistent across time points (Figures 4D and 4E). Biochemical analysis revealed comparable levels of both ALT and AST, indicating excellent hepatic safety (Figure S4C). Collectively, these results demonstrate that GA-28c exerts a durable, well-tolerated silencing effect suitable for long-term dosing strategies.

We next evaluated its therapeutic efficacy in a high-fat diet (HFD)-induced MASLD mouse model. Male C57BL/6 mice were fed an HFD for 6 weeks, followed by subcutaneous GA-28c injections (2.5 mg/kg) every 2 weeks for 10 weeks (Figure 4F). Upon the 11 week after first dose, mice were sacrificed and tissues were collected. RT-qPCR and western blot confirmed robust suppression of *Acsl4* (84.69%, $p < 0.001$) at both mRNA and protein levels in the liver (Figures 4G and 4H), with no abnormal elevation in ALT or AST (Figure 4I). Histological analyses demonstrated marked improvements in hepatic steatosis: Compared with HFD-controls, H&E staining showed the GA-28c-treated mice preserved hepatocyte structure, and oil red O (ORO) staining revealed a 33.12% reduction in lipid droplet accumulation ($p < 0.0001$) (Figure 4J), recapitulating the effects as previously documented in *Acsl4* KO mice.[64]

To our surprise, GA-28c treatment revealed unexpected benefits in serum lipid profiling. The GA-28c treatment normalized HFD-induced dyslipidemia, restoring total cholesterol (T-CHO), high-density lipoprotein cholesterol (HDL-C), and LDL-C levels toward those of standard diet controls (reductions of 69.17%, 49.34%, and

**Figure 4. *In vivo* assessment of GalNAc conjugates for liver-targeted *Acsl4* silencing and therapeutic efficacy of GA-28c in a HFD-induced MASLD mouse model**

(A) Schematic illustration of the free-uptake experiment in primary mouse hepatocytes used to assess the cellular activity of GalNAc-ASO conjugates. PMC, primary mouse hepatocytes cell. (B) Dose-dependent knockdown of *Acsl4* mRNA in primary mouse hepatocytes following free uptake (48 h) of GA-28c as measured by RT-qPCR with *Gapdh* as internal control. Data are presented as mean ± SD; $n = 3$ biological replicates. (C) Cartoon showing the *in vivo* assessment in mouse model for GA-28c silencing (upper) and western blot analysis of *Acsl4* protein expression in liver and kidney tissues from mice treated with GA-28c (low). (D) Cartoon showing the administration plan of GA-28c in mice (C57BL/6) by subcutaneous injection (upper) and western blot analysis of *Acsl4* protein expression in mouse liver tissues after 28 days (low). (E) Barplots showing *Acsl4* mRNA levels in mouse liver tissues over the 28-day time course, quantified by RT-qPCR with *Gapdh* as internal control. Data are presented as mean ± SD; $n = 3$ biological replicates. (F) Schematic diagram of the experimental procedure for HFD-induced MASLD modeling and GA-28c treatment. Male C57BL/6 mice were fed an HFD for 6 weeks followed by subcutaneous injections of GA-28c (2.5 mg/kg) every 2 weeks for 10 weeks. Tissues were collected at the endpoint (11 week after the first dose). (G) Western blot analysis of *Acsl4* protein expression in liver tissues at the study endpoint. (H) Barplots showing *Acsl4* mRNA levels in liver tissues at the endpoint, as determined by RT-qPCR with *Gapdh*as internal control. Data are presented as mean ± SD; $n = 5$ biological replicates. (I) Barplots showing ALT and AST levels measured at the endpoint (11 week). Data are presented as mean ± SD; $n = 5$ biological replicates. (J) Representative images of H&E and ORO staining of liver sections at the endpoint (left) and quantitative analysis of the lipid droplet area based on ORO staining. Data are presented as mean ± SD; $n = 5$ biological replicates. (K and L) Serum lipid profile at 5 weeks (F) and 11 week (G) after the first dose. Data are presented as mean ± SD; $n = 5$. For (B), (E), and (H)–(L), Statistical significance was determined by one-way ANOVA: n.s.: no significance, $^{*}p < 0.05$, $^{**}p < 0.01$, $^{***}p < 0.001$, $^{****}p < 0.0001$.

66.03%, respectively; all $p < 0.0001$) (Figures 4K and 4L). Given that dyslipidemia and MASLD is tightly correlated, these findings indicate GA-28c as a promising long-acting ASO therapeutic for MASLD, especially in obese patients.

### GA-28c treatment suppresses lipid synthesis and enhances triglyceride metabolism

To explore the molecular basis of these effects, we performed RNA sequencing (RNA-seq) of the liver tissues. Gene set enrichment analysis (GSEA) revealed that GA-28c significantly suppressed the fatty acid metabolism pathway (NES = −1.43), indicating inhibition of hepatic lipid synthesis (Figure S5A). Moreover, Gene Ontology (GO) analysis of downregulated genes also showed strong enrichment in lipid metabolic processes (Figure S5B). Examination of individual genes with RT-qPCR further confirmed widespread downregulation of multiple key regulators in fatty acid metabolism (Figures S5C and S5D).

In addition to suppressing lipid synthesis pathways, GA-28c treatment significantly enhanced the triglyceride metabolic process (Figures S5E and S5F). Importantly, genes related to lipid transport and clearance, such as *Pik3cg* and *Sorl1*, were markedly upregulated, a finding corroborated by RT-qPCR validation (Figures S5G and S5H). Together, these results suggest that GA-28c not only inhibits hepatic lipid accumulation but also promotes lipid turnover and clearance, contributing to improved systemic lipid homeostasis.

To assess potential off-target effects, transcriptomic analysis was conducted in HepG2 cells following GA-28c transfection. Out of 16,827 genes detected to express, only 6 genes were significantly downregulated, confirming high target specificity and minimal off-target activity (Figure S5I). Taking together, these data indicate that GA-28c exerts multifaceted metabolic benefits through precise suppression of the *ACSL4*-mediated fatty acid metabolism pathway, thereby reprogramming systemic lipid metabolism. This dual regulatory mechanism effectively alleviates hepatic steatosis and normalizes systemic lipid profiles in the MASLD model. Overall, these findings indicate GA-28c as a safe, liver-selective, and long-acting ASO therapeutic candidate with broad potential for treating metabolic liver disease and obesity-associated dyslipidemia.

## DISCUSSION

Owing to their sequence-specific mechanism, gapmer ASOs offer remarkable potential for precision and personalized medicine.[66] However, their development faces unique challenges due to the complexity of RNA biology and species variation. Since ASOs act primarily within the nucleus by targeting precursor mRNA, the vast number of possible targetable regions creates a significant obstacle for optimal sequence selection and functional validation.[26] While large pharmaceutical companies may perform exhaustive screening across the full gene to identify potent candidates, cross-species sequence divergence often limits translational validation—many lead ASOs designed for human targets fail to maintain efficacy in animal models. Moreover, humanized models frequently lack intronic regions, preventing accurate assessment of nuclear-targeting ASOs. Finally, single-nucleotide polymorphisms (SNPs) pose another critical challenge. Even a single mismatch can dramatically reduce silencing potency, introducing variability in therapeutic outcomes and potential inconsistencies during clinical translation.[32]

Here, we present ClinASO, an integrated computational-experimental platform that streamlines ASO discovery by incorporating multiple key determinants—sequence accessibility, SNP filtering, off-target prediction, RNase H1 cleavage preference, and cross-species conservation—into a unified design framework (Figure 1A). ClinASO efficiently identifies potent ASO candidates with compatible wild-type animal models for *in vivo* validation. Using *PCSK9* and *IRS1* as examples, we demonstrate that ClinASO can rapidly pinpoint highly potent sequences from a limited candidate pool, achieving superior silencing efficacy compared with benchmark therapeutics, such as Inclisiran and Aganirsen, both *in vitro* and *in vivo*.

To further validate the efficiency of ClinASO, we selected *ACSL4*, a recently identified but still controversial therapeutic target for MASLD,[64] for which no ASO therapy currently exists. Using ClinASO, we rapidly identified several potent sequences (Figures 3A and 3B), and through targeted optimization generated GA-28c, a 2′-cEt-modified, GalNAc-conjugated ASO enabling liver-specific delivery. In a high-fat diet (HFD)–induced MASLD mouse model, GA-28c produced robust therapeutic benefits. It markedly reduced hepatic lipid accumulation and unexpectedly restored systemic lipid balance, normalizing serum cholesterol and lipoprotein levels (Figures 4J–4L). Transcriptomic analyses revealed dual regulatory effects—suppression of fatty acid synthesis and activation of lipid clearance pathways—indicating a systems-level correction of dyslipidemia (Figure S5). Minimal off-target transcriptional changes further confirmed the precision of ClinASO-guided design (Figure S5I).

Importantly, GA-28c exhibited a long-acting pharmacodynamic profile, maintaining >60% target suppression for over 2 weeks after a single low-dose injection (2.5 mg/kg) (Figures 4D and 4E). Given the faster metabolic rate in mice, this suggests the potential for monthly or less-frequent dosing in humans—a major advantage over current MASLD therapies requiring daily administration. The normalization of HDL-C and LDL-C likely reflects restored physiological lipid flux rather than an atherogenic effect, consistent with enhanced hepatic lipid turnover.

It is of note that SNPs represent a critical determinant in ASO drug development, as even a single mismatch can dramatically reduce target binding or efficacy in certain patient populations.[34,67] However, such an important issue was often overlooked in early ASO drug development due to limited genomic data. Leveraging the rapidly expanding genomic databases, now encompassing ∼1.0 billion human SNPs across diverse subpopulations, ClinASO integrates comprehensive SNP annotation directly into its design pipeline

(Figure 1A). By systematically excluding polymorphic sites and prioritizing conserved regions, the platform effectively minimizes genotype-dependent variability and enhances clinical translatability of designed ASOs.

Collectively, these results demonstrate that ClinASO enables rapid identification and validation of potent, species-compatible ASO sequence candidates, thereby offering a powerful tool for accelerating ASO drug development and supporting the advancement of precision and personalized RNA therapeutics. However, it is important to note that empirical sequence walking is highly recommended for identifying optimal ASO sequences for further clinical development. Indeed, while factors such as secondary structure and cleavage efficiency can be approximated computationally, biological complexity often limit the predictive power of these approaches, which therefore does not exclude the possibility to have better candidates sequence.

Safety is also a critical consideration in drug development. Sequence-dependent off-target effects represent a major concern for oligonucleotide therapeutics, including ASOs. In the ClinASO algorithm, we implemented a BLAST-based strategy to identify potential off-target sites. However, unlike siRNAs that have well-defined seed regions to facilitate off-target prediction,[68] the sequence determinants governing gapmer ASO off-target interactions remain poorly defined. Therefore, experimental assessment of off-target effects is strongly recommended. In addition, toxicity could be derived from certain sequence motifs combined with certain modifications.[69] These forms of toxicity currently lack well-defined rules that would allow reliable computational prediction. Therefore, independent experimental evaluation of ASO toxicity remains a must during candidate development.

## MATERIALS AND METHODS

### Oligonucleotide synthesis and purification

Antisense oligonucleotides were synthesized and purified by SicaGene (Beijing, China).

### Oligonucleotides used in the study

Names and sequences of all the oligonucleotides used in the study are shown in Tables S1, S2, and S3.

### HepG2, Huh7,293 T, HUVEC, and Hepa1-6 cells culture

Upon reaching 80% confluence, the culture medium was aspirated and discarded. The cell layer was gently rinsed twice with sterile phosphate-buffered saline (PBS) to remove residual serum and metabolic waste. Following the removal of PBS, pre-warmed (37°C) 0.25% trypsin solution (Gibco Cat. 25200056) was added to cover the cell layer. The culture vessel was then incubated at 37°C in a 5% $CO_2$ atmosphere for 3 min to facilitate dissociation. The enzymatic reaction was promptly terminated by adding an equal volume of DMEM (Gibco Cat. 11965092) complete medium (supplemented with 10% fetal bovine serum [FBS], (Procell Cat. 164210). The cells were subsequently detached by gentle pipetting to generate a single-cell suspension. This suspension was transferred to a sterile centrifuge tube and centrifuged at 1,000 $\times$ $g$ for 3 min. After centrifugation, the supernatant was carefully aspirated and discarded. The cell pellet was resuspended in a suitable volume of pre-warmed DMEM complete medium. Finally, the cell suspension was seeded into new culture vessels at an appropriate split ratio (1:3), supplemented with fresh medium, and incubated at 37°C under 5% $CO_2$ for continued growth.

### ASO transfection (HepG2,293 T, Hepa1-6, Huh7)

Log-phase cells were seeded in 12-well plates at a density of $1.65 \times 10^5$ cells/well with 1 mL of DMEM (Gibco Cat. 11965092) supplemented with 10% FBS. After 24 h of incubation at 37°C under 5% $CO_2$, when cells reached 70% confluence, the medium was replaced with serum-free DMEM. ASO stock solutions were prepared at concentrations of 2 μM, and 0.2 μM using nuclease-free water. Working solutions (0, 5, 15, 44 nM) were subsequently diluted from the stocks with nuclease-free water. For transfection complex formation, 12.5 μL of Each ASO working solution was mixed with 25 μL of $CaCl_2$ solution (Beyotime Cat. C0508). The mixture was vortexed briefly, followed by the addition of 25 μL BBS buffer (Beyotime Cat. C0508). After vortex mixing for 5 s, the solution was incubated at room temperature for 15 min. The resulting complexes (62.5 μL/well) were added dropwise to the plates, which were gently swirled to ensure uniform distribution, and incubated for 8 h at 37°C. Subsequently, the transfection medium was replaced with DMEM containing 10% FBS. Cells were harvested at 12 h post-transfection for RNA analysis or at 48 h for protein extraction.

### ASO transfection (HUVEC cells)

For ASO transfection in HUVECs using Lipofectamine RNAiMAX, we followed the manufacturer's protocol with slight modifications. Briefly, HUVECs were seeded in antibiotic-free growth medium at 35,000 cells per well in a 24-well plate one day prior to transfection to achieve 30%–50% confluence. For each well, ASO was diluted in 50 μL of Opti-MEM I Reduced Serum Medium (Thermo Fisher Scientific, Cat. 31985–062). Separately, 1 μL of Lipofectamine RNAiMAX reagent (Thermo Fisher Scientific, Cat. 13778–150) was diluted in 50 μL of Opti-MEM I Medium. The diluted ASO was combined with the diluted reagent, incubated for 10–20 min at room temperature to form complexes, and then added to the cells. The cells were incubated with the complexes at 37°C in a $CO_2$ incubator, and gene knockdown was analyzed 24 h post-transfection.

### RT-qPCR

Total RNA was extracted from samples using TriQuick Reagent (Solarbio Cat. R1100) according to the manufacturer's protocol. Briefly, samples were homogenized in TriQuick Reagent, mixed with chloroform, and centrifuged at 15,000 $\times$ $g$ for 15 min at 4°C. The aqueous phase was collected, and RNA was precipitated using sodium acetate (pH 5.2) and isopropanol. The resulting RNA pellets were washed with 75% ethanol, air-dried, and dissolved in DEPC-treated water. RNA purity was assessed spectrophotometrically, with A260/A280 ratios between 1.8 and 2.0 considered acceptable, and concentrations

were measured to ensure values within the range of 100–200 ng/μL. Complementary DNA (cDNA) was synthesized from 500 ng of total RNA using the HiScript II Q RT SuperMix for qPCR (Vazyme Cat. R223-01), which includes reverse transcriptase and genomic DNA removal components, in a 20 μL reaction volume under the following conditions: 50°C for 15 min and 85°C for 5 min. Quantitative real-time PCR was performed using PerfectStart Green qPCR SuperMix (TransGen Cat. AQ601) in a 384-well plate format on LightCycler 480 II (Roche). Each 15 μL reaction contained 7.5 μL of SuperMix, 0.3 μL of each gene-specific primer (10 μM), and 5 μL of cDNA template. The reaction conditions were as follows: initial denaturation at 95°C for 2 min; 40 cycles of denaturation at 95°C for 15 s, and annealing/extension at 60°C for 15 s (two-step method). Melting curve analysis was performed to confirm amplification specificity. Gene expression levels were normalized to species-specific internal control genes as follows: for *ACSL4* and *PCSK9*, *GAPDH* was used for human and mouse samples; for *SRPK1* and *IRS1*, *β-Actin* was used for human, mouse, and rabbit samples. Relative quantification was calculated using the $2-\Delta\Delta Ct$ method. *ACSL4* gene: Human: forward 5′–TGTGGACAATAAGGCTATCA–3′, reverse 5′–TGGTCTACTTGGAGGAATG–3′. Mouse: forward 5′–TTTGGCTCATGTGCTGGAAC–3′, reverse 5′–TCACCCTTGCTT CCCTTCTT–3′. *PCSK9* gene: Human: forward 5′–CAGAGTGAC CACCGGGAAAT–3′, reverse 5′–GTCACACTTGCTGGCCTG TC–3′. *SRPK1* gene: Human: forward 5′–GCAACAGAATGGCAG CGATC–3′, reverse 5′–CTGGCGCTTCTGCTTCTTC–3′.Mouse: forward 5′–AGCCTCATTCAGGGGAGGAT–3′, reverse 5′–TGAT GTGCTTCAGGTCACCTTT–3′. *IRS1* gene: Human: forward 5′–TCAAGTGAGGATTTAAGCGCC–3′, reverse 5′–ACTGAAATGG ATGCATCGTACC–3′. Mouse: forward 5′–AATTGCTCAGCTC CTCCTCA–3′, reverse 5′–GGCTCCACTTCAGACTGTCT–3′. Rabbit: forward 5′–AGGGCTGTGAGCAGATGAAC–3′, reverse 5′–TCTGAAGCCAAAGAGCCCTG–3′. Internal control genes: *GAPDH* (for *ACSL4* in human and mouse): Human: forward 5′–GTCAGCCGCATCTTCTTTTG–3′, reverse 5′–GCGCCCAATACG ACCAAATC–3′.*Vinculin*: Mouse: forward 5′–CGCTGGCGTCC ATAGACTC–3′, reverse 5′–CCTAGCATTTTGCAGGTTCCTA–3′. *β-Actin* (for *SRPK1* and *IRS1* in human, mouse, and rabbit): Human: forward 5′–CACCATTGGCAATGAGCGGTTC–3′, reverse 5′–AGGTCTTTGCGGATGTCCACGT–3′. Mouse: forward 5′–CA GCCTTCCTTCTTGGGTATG–3′, reverse 5′–GGCATAGAGGTCT TTACGGATG–3′. Rabbit: forward 5′–GGACATCAAGGAGAAG CTGTG–3′, reverse 5′–GGCAGCTCGTAGCTCTTCTC–3′.

## Western blotting

Cell or tissue samples were washed with pre-cooled PBS, then lysed on ice for 30 min using RIPA lysis buffer (50 mM Tris-HCl, pH 7.4; 150 mM NaCl; 1% NP-40, 0.5% sodium deoxycholate, PMSF 1 mM, and 0.1% SDS) containing protease inhibitors. The lysate was centrifuged at 12,000 × *g* for 10 min at 4°C to collect the supernatant. Protein concentration was determined using the BCA method (BCA Protein Assay Kit, Solarbio Cat. PC0020). Protein samples were mixed with 5× loading buffer and denatured by boiling at 100°C for 5 min. According to the molecular weight of the target protein, an SDS-PAGE gel was prepared and 30 μg of protein per lane was loaded. Electrophoresis was performed at constant voltage: 80 V in the stacking gel and 120 V in the separation gel, until the bromophenol blue reached the bottom of the gel. The PVDF membrane (Millipore Cat. IPVH00010) was activated in methanol for 1 min and equilibrated for 10 min in transfer buffer containing 20% methanol together with the gel. The transfer sandwich was assembled, ensuring all bubbles were removed. Transfer was carried out at constant voltage in an ice bath. The membrane was blocked with 5% skim milk/TBST at room temperature for 1 h, incubated with primary antibody (diluted 1:1,000) at 4°C overnight, washed 3 times with TBST, then incubated with secondary antibody (diluted 1:10,000) at room temperature for 1 h, and finally washed 3 times with TBST (15 min each). ECL chemiluminescence reagents A and B (Solarbio Cat. PE0010) were mixed in equal volumes, applied to cover the membrane surface, and signals were captured using a chemiluminescence imaging system. The following primary antibodies were used in the experiments: anti- ACSL4 (Santa Cruz Biotechnology, Cat. sc-365230), anti-GAPDH (Santa Cruz Biotechnology, Cat. sc-32233).

## Lipid droplet staining in cells

Cells were seeded into 12-well cell culture plates containing pre-sterilized coverslips and treated with 0.25 mM oleic acid to establish an MASLD cellular model.[70] After 24 h, ASOs were delivered into the cells via calcium phosphate-mediated transfection. Forty-eight hours post-transfection, the culture medium was aspirated and cells were washed gently with PBS. Subsequently, cells were stained with BODIPY (MCE Cat. HY-D1614) and Hoechst (MCE Cat. HY-15627) at a dilution of 1:20,000 for 10 min under light-protected conditions. After removal of the staining solution, cells were washed 3 times with PBS (5 min per wash). Fixation was performed using 4% paraformaldehyde for 10 min at room temperature, followed by three additional PBS washes. Coverslips were mounted onto glass slides using an appropriate mounting medium and imaged by confocal microscopy.

## Isolation and free uptake assay of primary mouse hepatocytes

Neonatal mice C57BL/6 J aged 8–10 days were euthanized by cervical dislocation. The abdominal skin and muscle layers were transversely incised, and the liver was gently extruded from the abdominal cavity. The intact liver tissue was excised and rinsed in PBS to remove blood contaminants. The washed liver was transferred to a sterile culture dish, minced into a paste-like consistency (approximately 1 $mm^3$ fragments) using sterile ophthalmic scissors, and rinsed with 3 mL of Hanks' buffer via gentle pipetting. The tissue fragments were filtered twice through a 70 μm cell strainer to remove connective tissues. The filtered tissue was resuspended in 3 mL of Hanks' buffer, followed by the addition of 60 μL of collagenase I (1 mg/mL) (Yeasen Cat. 40507ES60) and 60 μL of collagenase IV (1 mg/mL) (Yeasen Cat. 40510ES60). The mixture was incubated at 37°C for 15 min, gently pipetted to homogenize, and digested for an additional 15 min. After digestion, the suspension was filtered through a 70 μm strainer into a 50 mL centrifuge tube and centrifuged at 1,000 × *g* for 3 min. The supernatant was discarded, and the pellet was resuspended in

DMEM high-glucose medium supplemented with 10% FBS. The cell suspension was seeded into a 15 cm culture dish, mixed thoroughly using a cross-shaped pattern, and cultured at 37°C under 5% $CO_2$. The medium was replaced every 3 days for a total of 7 days. After cultivation, the old medium was aspirated, and cells were detached with 0.25% trypsin for 2 min. The digestion reaction was neutralized with DMEM medium containing serum. The cell suspension was centrifuged at 1,000 $\times g$ for 3 min, and the pellet was resuspended in DMEM medium with 10% FBS. Cells were seeded into a 12-well plate at a density of $5 \times 10^5$ cells per well and cultured for 24 h. ASOs were directly added to the culture, followed by 48 h of incubation. Changes in protein and mRNA expression levels were subsequently analyzed.

### *In vitro* angiogenesis assay

Matrigel (Dow Corning Cat. 354230) was thawed at 4°C overnight and maintained on ice throughout the experiment to prevent premature polymerization. A pre-cooled 96-well plate was placed on ice and 50 μL Matrigel was carefully dispensed into each well with pre-cooled pipette tips to ensure uniform coating. The plate was then moved to a 37°C incubator for 1 h to allow solidification. HUVECs were detached using 0.25% trypsin-EDTA (Gibco Cat. 25200056), harvested by centrifugation at 1,000 $\times$ $g$ for 3 min and resuspended in complete Endothelial Cell Medium (ECM, ScienCell Cat. 1001). The cell concentration was adjusted to $1.5 \times 10^6$ cell/mL. One milliliter of the cell suspension was distributed evenly in ten sterile 1.5 mL microcentrifuge tubes for a final concentration of $1.5 \times 10^4$ cells per tube. Appropriate amounts of ASOs were added to the cell suspension in each tube, which was mixed gently by pipetting to ensure good contact between ASO and cells. Then the tubes were incubated for 10 min at room temperature to allow sufficient interaction between ASO and cells. After the pre-incubation, 100 μL of the ASO-cell suspension ($1.5 \times 10^4$ cells) was seeded gently in each Matrigel-coated well and placed in a humidified incubator at 37°C at 5% $CO_2$ for 24 h to allow for tubule formation. After a 24-h incubation, 1 μg/μL Calcein AM (Invitrogen Cat. 65085378) was mixed with DMEM at a ratio of 1:50 to prepare a working solution. Then the culture medium was carefully aspirated and replaced with 50 μL of Calcein AM working solution per well. The plate was incubated at room temperature for 30 min in the dark. The wells were washed twice with pre-warmed PBS to remove any remaining dye following the staining procedure. Tube-like structures were visualized and imaged under a fluorescence microscope equipped with a fluorescein isothiocyanate (FITC) filter set.

### Animal experiments

All animal procedures were conducted in accordance with the ethical standards of the Laboratory Animal Center of Yunnan University and approved by its Animal Care and Use Committee (approval nos.: YNU20251623 and YNU20251598). Experiments adhered strictly to institutional guidelines for the care and use of laboratory animals. All efforts were made to minimize animal suffering, and euthanasia was performed using methods consistent with the AVMA Guidelines for the Euthanasia of Animals.

### *In vivo* validation of *SRPK1* ASO

Adult male C57BL/6 mice (6–8 weeks old) were randomly assigned to treatment and control groups. A candidate ASO targeting *SRPK1* (100 μg in 1 μL saline) was administered via intravitreal injection into the right eye under appropriate anesthesia. Control mice received an equal volume of saline vehicle via the same route. Three days post-injection, animals were euthanized, and whole eyes were harvested immediately for total RNA extraction. Knockdown efficiency of *Srpk1* was quantified using RT-qPCR.

### *In vivo* validation of *IRS1* ASO

For systemic evaluation, juvenile New Zealand White rabbits (approximately 200 g) received intraperitoneal injections of *IRS1*-targeting ASOs at a dose of 30 mg/kg. For ocular delivery, adult New Zealand White rabbits (approximately 2 kg) were administered a subconjunctival injection of I-17 ASO (1 mg in 40 μL) into the right eye, while the contralateral eye received a scrambled ASO control. Animals were housed under a 12-h light/dark cycle with ad libitum access to food and water. After 72 h, rabbits were euthanized and tissue samples were collected.

### Duration of *ACSL4* ASO effect

To evaluate the duration of ASO-mediated gene silencing, 8-week-old C57BL/6 mice were administered a single dose of *Acsl4*-targeting ASO. Animals were euthanized at predetermined time points post-injection. Liver tissues were collected to determine the persistence of *ACSL4* knockdown via RT-qPCR analysis. Concurrently, blood samples were collected at each time point for hematological and biochemical analyses to monitor systemic toxicity.

### Therapeutic efficacy of *ACSL4* ASO in a HFD model

4-week-old C57BL/6 mice were fed an HFD (60 kcal% fat, Synergy Bio Cat. XTHF60) for 6 weeks to establish a metabolic disorder model. Mice received subcutaneous injections of GA-28c every 2 weeks for a total of 5 doses. Control animals received saline injections on the same schedule. One week after the final treatment, mice were euthanized, and blood along with relevant tissues was harvested for evaluation of therapeutic efficacy.

### Serum biochemical analysis

Blood was collected via retro-orbital plexus under isoflurane anesthesia. Serum was isolated by centrifugation at 1,000 $\times g$ for 15 min after 30-min clotting at room temperature. Triglycerides (TG), total cholesterol (TC), HDL-C, and LDL-C were measured using enzymatic kits (Nanjing Jiancheng Bioengineering, Cat. A110-1-1, Cat. A111-1-1, Cat. A112-1-1, Cat. A113-1-1). ALT and AST were analyzed with kits (Nanjing Jiancheng Bioengineering Cat. C009-2-1, Cat. C010-2-1).

### Histopathological analysis

#### *H&E staining*

Tissue blocks were fixed in 4% paraformaldehyde for 24 h, followed by gradient dehydration through 75%, 85%, 95%, and 100% ethanol. After dehydration, the tissues were cleared in xylene for 30 min and

infiltrated with molten paraffin wax (replaced every 20 min, repeated twice) for a total of 2–3 h, then embedded and cooled. Sections of 5 μm thickness were cut, expanded in 42°C warm water, mounted on slides, and baked in a 65°C oven for 30 min. After deparaffinization in xylene (2 × 10 min) and rehydration through an ethanol gradient (100% → 75%, 10 min each step), the sections were processed as follows: For H&E staining, nuclei were stained with Hematoxylin (Solarbio Cat. G1120) for 2 min, rinsed under running water, differentiated in 0.5% hydrochloric acid ethanol for 30 s, blued in running water, and cytoplasm was stained with Eosin (Solarbio Cat. G1120) for 3 min, followed by gradient ethanol dehydration, xylene clearing, and mounting with neutral balsam.

#### *ORO staining*

Fresh liver tissue was embedded in O.C.T. compound (Tissue-Tek Cat. 4583) and rapidly frozen at −80°C. The frozen tissue was sectioned at 8 μm thickness using a cryostat maintained at −20°C, and the sections were mounted on glass slides. After warming at room temperature for 10 min, the ORO (Sigma Cat. C.I.26125) working solution was prepared immediately before use by mixing the stock solution with distilled water at a 3:2 ratio, filtered through a 0.45 μm membrane filter, and allowed to stand protected from light for 10 min. The filtered solution was applied to fully cover the tissue sections, which were then incubated at room temperature in the dark for 5 min. After staining, the sections were rinsed in water for 20 min. The sections were mounted with glycerol gelatin and imaged immediately using a digital slide scanner to prevent fading of lipid droplets.

### RNA-seq and bioinformatics

After total RNA extraction, mRNA was enriched from the purified total RNA using the Vazyme mRNA Capture Beads (Vazyme Cat. N401-02). Libraries were then constructed with the Vazyme Universal V8 RNA-seq Library Prep Kit for Illumina (Vazyme Cat. NR605-02). Sequencing was performed on a SURFSeq 5000 platform (Genemind Biotechnology Co., Ltd.) to generate 100-bp single-end reads. Raw sequencing reads were quality controlled and adapter trimmed using Trim Galore (v.0.6.10). The cleaned reads were aligned to the *Mus musculus* or *Homo sapiens* reference genome (GRCm39/GRCh38.p14) with STAR (v.2.7.11 b),[71] and gene expression quantification was performed using featureCounts (v.1.6.3).[72] Differential expression analysis was conducted with DESeq2 (v.1.44.0),[73] applying thresholds of $|\log_2 \text{fold change}| > 1$ and adjusted $p$ value $<0.05$. Significantly differentially expressed genes were subsequently subjected to GO enrichment analysis using clusterProfiler (v.4.12.6)[74] with a significance cutoff of $p < 0.01$.

### Statistical analysis

Unless otherwise noted, results are presented as mean ± standard deviation (SD). For comparisons involving four or more groups, one-way analysis of variance (ANOVA) followed by multiple comparison *t* tests was performed to assess statistical significance. Results are presented as mean ± standard deviation (SD). Statistical significance was defined as follows: n.s., not significant, $^*p < 0.05$, $^{**}p < 0.005$, $^{***}p < 0.0005$, and $^{****}p < 0.0001$.

### ClinASO web platform implementation

ClinASO was implemented as a web-based platform using standard front-end and back-end technologies, with both the client-side and server-side source code publicly available on GitHub. The front end was developed using HTML5, CSS3, and JavaScript, providing a responsive and interactive user interface. The platform interface is organized into six major functional modules, including ASO introduction and mechanism, gapmer design, homology analysis, SNP analysis, off-target analysis, and reference/contact information. These modules are integrated through a tab-based navigation system, enabling users to switch efficiently among different analytical functions.

To support interactive analysis, the front end adopted a unified asynchronous form submission mechanism. After a form is submitted, user-provided parameters are collected and transmitted to the back-end server, while the interface dynamically updates the processing status and disables repeated submission during task execution. Once the server returns the analysis result in JSON format, the corresponding status or output information is rendered on the page without requiring a full refresh. For example, in the gapmer design module, parameters such as gene name and ASO length are submitted to the designated back-end endpoint, and the returned progress or completion message is displayed to the user. This design improves usability and responsiveness of the platform.

The back end of ClinASO was constructed using the Python Flask framework, with the core application logic integrated into the main application file. Cross-origin resource sharing (CORS) was enabled to ensure seamless communication between the front-end and back-end components. The routing system supports four major analytical workflows, including gapmer design, homology analysis, SNP analysis, and off-target analysis, each of which is handled by an independent request-processing function.

The computational workflows were implemented by invoking external Bash scripts through Python's subprocess module. For example, the gapmer design workflow calls a shell script that further invokes downstream Python modules for sequence analysis and optimization. To ensure task isolation and avoid interference among concurrent jobs, a unique temporary working directory is generated for each task. Analysis outputs, including Excel spreadsheets and PDF reports, are stored in the corresponding task-specific output directory.

After task completion, temporary files are automatically removed to reduce unnecessary disk usage and maintain a clean execution environment. In addition, ClinASO integrates an SMTP-based email notification system for automated result delivery. According to the execution status of each task, the platform can send HTML-formatted email notifications indicating successful completion, execution errors, or timeout events. Together, these design features improve the robustness, usability, and practicality of ClinASO for ASO-related analyses.

## DATA AND CODE AVAILABILITY

All RNA-seq data were submitted to the Gene Expression Omnibus with ID GSE307517.

## ACKNOWLEDGMENTS

This work was supported by the National Natural Science Foundation of China (32470609 and 32171262 to Y.D., 32460138 to J.Y, 32570663 and 32070626 to F.L., and 32560140 to G.R.), Beijing MSTC (Z231100004823024 to H.W.), the Open Research Program of State Key Laboratory for Conservation (2023KF009 to Y.D.), Yunnan Province Science and Technology Department (202401BF070001-013 to Y.D., 202301AT070083 and 202401AT070019 to J.Y., 202201BF070001-015 to F.L., and 202501AS070057 and 202401AT070436 to G.R.). National Key Research and Development Program of China (2025YFC3409700 to K.Z.), and Guangdong Basic and Applied Basic Research Foundation (2023B1515020109 to K.Z.). We acknowledge the Dang and Lai lab members for helping sequencing works.

## AUTHOR CONTRIBUTIONS

Y.D., F.L., and H.W. conceived and supervised the overall project. S.C., H.L., and D.C. preformed most of the experiments. S.C. performed most bioinformatics analysis. Y.W., H.W., F.L., K.Z., and J.Y. helped the design and synthesis of ASOs. A.D., J.B., and S.G. participated in assays in human cells. J.B. and S.G. also participated in the library construction and sequencing of RNA-seq. Y.D., F.L., and S.C. wrote the manuscript with critical feedback from all co-authors.

## DECLARATION OF INTERESTS

H.W., F.L., X.W., and Y.D. are co-founders of SicaGene BioScience Co., Ltd. H.W., F.L, X.W., Y.D., D.C., and Y.W. are inventors on patent applications related to GalNAc conjugation chemistry and tested ASO sequences (including *IRS*-, *PCSK9*-, and *SRPK1*-targeting ASOs). The ClinASO platform and associated software are released as open-source under the MIT License.

## SUPPLEMENTAL INFORMATION

Supplemental information can be found online at https://doi.org/10.1016/j.omtn.2026.102933.

## REFERENCES


1. Stephenson, M.L., and Zamecnik, P.C. (1978). Inhibition of Rous sarcoma viral RNA translation by a specific oligodeoxyribonucleotide. Proc. Natl. Acad. Sci. USA *75*, 285–288.
2. Crooke, S.T., Baker, B.F., Crooke, R.M., and Liang, X.-h. (2021). Antisense technology: an overview and prospectus. Nat. Rev. Drug Discov. *20*, 427–453.
3. Roberts, T.C., Langer, R., and Wood, M.J.A. (2020). Advances in oligonucleotide drug delivery. Nat. Rev. Drug Discov. *19*, 673–694.
4. Boiziau, C., Kurfurst, R., Cazenave, C., Roig, V., Thuong, N.T., and Toulmé, J.J. (1991). Inhibition of translation initiation by antisense oligonucleotides via an RNase-H independent mechanism. Nucleic Acids Res. *19*, 1113–1119.
5. Hammond, S.M., Aartsma-Rus, A., Alves, S., Borgos, S.E., Buijsen, R.A.M., Collin, R.W.J., Covello, G., Denti, M.A., Desviat, L.R., Echevarría, L., et al. (2021). Delivery of oligonucleotide-based therapeutics: challenges and opportunities. EMBO Mol. Med. *13*, e13243.
6. Crooke, S.T., Wang, S., Vickers, T.A., Shen, W., and Liang, X.H. (2017). Cellular uptake and trafficking of antisense oligonucleotides. Nat. Biotechnol. *35*, 230–237.
7. Chen, S., Heendeniya, S.N., Le, B.T., Rahimizadeh, K., Rabiee, N., Zahra, Q.U.A., and Veedu, R.N. (2024). Splice-Modulating Antisense Oligonucleotides as Therapeutics for Inherited Metabolic Diseases. BioDrugs *38*, 177–203.
8. Sumner, C.J., and Miller, T.M. (2024). The expanding application of antisense oligonucleotides to neurodegenerative diseases. J. Clin. Investig. *134*, e186116.
9. Schoch, K.M., and Miller, T.M. (2017). Antisense Oligonucleotides: Translation from Mouse Models to Human Neurodegenerative Diseases. Neuron *94*, 1056–1070.
10. Banadaki, H.D., Garanto, A., and Collin, R.W.J. (2025). Antisense oligonucleotides for inherited retinal diseases: a comprehensive review. Mol. Aspect. Med. *106*, 101416.
11. Xue, K., and MacLaren, R.E. (2020). Antisense oligonucleotide therapeutics in clinical trials for the treatment of inherited retinal diseases. Expet Opin. Invest. Drugs *29*, 1163–1170.
12. Christensen, J.K., Colletti, N., Hooshfar, S., Jiang, R.R., Kuo, C., Lindmark, B., Lumen, A., Youssef, A.S., Albertolle, M., Aluri, K.C., et al. (2025). Translational and clinical development of therapeutic siRNA and ASOs: current industry practices, perspectives, and recommendations. Nucleic Acids Res. *53*, gkaf778.
13. Sang, A., Zhuo, S., Bochanis, A., Manautou, J.E., Bahal, R., Zhong, X.B., and Rasmussen, T.P. (2024). Mechanisms of Action of the US Food and Drug Administration-Approved Antisense Oligonucleotide Drugs. BioDrugs *38*, 511–526.
14. Monia, B.P., Lesnik, E.A., Gonzalez, C., Lima, W.F., McGee, D., Guinosso, C.J., Kawasaki, A.M., Cook, P.D., and Freier, S.M. (1993). Evaluation of 2‘-modified oligonucleotides containing 2‘-deoxy gaps as antisense inhibitors of gene expression. J. Biol. Chem. *268*, 14514–14522.
15. Geary, R.S., Norris, D., Yu, R., and Bennett, C.F. (2015). Pharmacokinetics, biodistribution and cell uptake of antisense oligonucleotides. Adv. Drug Deliv. Rev. *87*, 46–51.
16. Wu, H., Lima, W.F., Zhang, H., Fan, A., Sun, H., and Crooke, S.T. (2004). Determination of the role of the human RNase H1 in the pharmacology of DNA-like antisense drugs. J. Biol. Chem. *279*, 17181–17189.
17. Eckstein, F. (2014). Phosphorothioates, essential components of therapeutic oligonucleotides. Nucleic Acid Therapeut. *24*, 374–387.
18. Gaus, H.J., Gupta, R., Chappell, A.E., Østergaard, M.E., Swayze, E.E., and Seth, P.P. (2019). Characterization of the interactions of chemically-modified therapeutic nucleic acids with plasma proteins using a fluorescence polarization assay. Nucleic Acids Res. *47*, 1110–1122.
19. Koralewska, N., Corradi, E., Milewski, M.C., Masante, L., Szczepanska, A., Kierzek, R., Figlerowicz, M., Baudet, M.L., and Kurzynska-Kokorniak, A. (2024). Short 2’-O-methyl/LNA oligomers as highly-selective inhibitors of miRNA production in vitro and in vivo. Nucleic Acids Res. *52*, 5804–5824.
20. Freier, S.M., and Altmann, K.H. (1997). The ups and downs of nucleic acid duplex stability: structure-stability studies on chemically-modified DNA:RNA duplexes. Nucleic Acids Res. *25*, 4429–4443.
21. Wan, W.B., and Seth, P.P. (2016). The Medicinal Chemistry of Therapeutic Oligonucleotides. J. Med. Chem. *59*, 9645–9667.
22. Shen, W., De Hoyos, C.L., Migawa, M.T., Vickers, T.A., Sun, H., Low, A., Bell, T.A., III, Rahdar, M., Mukhopadhyay, S., Hart, C.E., et al. (2019). Chemical modification of PS-ASO therapeutics reduces cellular protein-binding and improves the therapeutic index. Nat. Biotechnol. *37*, 640–650.
23. Nair, J.K., Willoughby, J.L.S., Chan, A., Charisse, K., Alam, M.R., Wang, Q., Hoekstra, M., Kandasamy, P., Kel’in, A.V., Milstein, S., et al. (2014). Multivalent N-acetylgalactosamine-Conjugated siRNA Localizes in Hepatocytes and Elicits Robust RNAi-Mediated Gene Silencing. J. Am. Chem. Soc. *136*, 16958–16961.
24. Prakash, T.P., Graham, M.J., Yu, J., Carty, R., Low, A., Chappell, A., Schmidt, K., Zhao, C., Aghajan, M., Murray, H.F., et al. (2014). Targeted delivery of antisense oligonucleotides to hepatocytes using triantennary N-acetyl galactosamine improves potency 10-fold in mice. Nucleic Acids Res. *42*, 8796–8807.
25. Ashwell, G., and Harford, J. (1982). Carbohydrate-specific receptors of the liver. Annu. Rev. Biochem. *51*, 531–554.
26. Lai, F., Damle, S.S., Ling, K.K., and Rigo, F. (2020). Directed RNase H Cleavage of Nascent Transcripts Causes Transcription Termination. Mol. Cell *77*, 1032–1043.e4.
27. Pedersen, L., Hagedorn, P.H., Lindholm, M.W., and Lindow, M. (2014). A Kinetic Model Explains Why Shorter and Less Affine Enzyme-recruiting Oligonucleotides Can Be More Potent. Mol. Ther. Nucleic Acids *3*, e149.
28. Vickers, T.A., Wyatt, J.R., and Freier, S.M. (2000). Effects of RNA secondary structure on cellular antisense activity. Nucleic Acids Res. *28*, 1340–1347.
29. Hagedorn, P.H., Pontoppidan, M., Bisgaard, T.S., Berrera, M., Dieckmann, A., Ebeling, M., Møller, M.R., Hudlebusch, H., Jensen, M.L., Hansen, H.F., et al. (2018). Identifying and avoiding off-target effects of RNase H-dependent antisense oligonucleotides in mice. Nucleic Acids Res. *46*, 5366–5380.

30. Hörberg, J., Carlesso, A., and Reymer, A. (2024). Mechanistic insights into ASO-RNA complexation: Advancing antisense oligonucleotide design strategies. Mol. Ther. Nucleic Acids *35*, 102351.

31. Kielpinski, L.J., Hagedorn, P.H., Lindow, M., and Vinther, J. (2017). RNase H sequence preferences influence antisense oligonucleotide efficiency. Nucleic Acids Res. *45*, 12932–12944.

32. Magner, D., Biala, E., Lisowiec-Wachnicka, J., and Kierzek, R. (2017). Influence of mismatched and bulged nucleotides on SNP-preferential RNase H cleavage of RNA-antisense gapmer heteroduplexes. Sci. Rep. *7*, 12532.

33. Yasuhara, H., Yoshida, T., Sasaki, K., Obika, S., and Inoue, T. (2022). Reduction of Off-Target Effects of Gapmer Antisense Oligonucleotides by Oligonucleotide Extension. Mol. Diagn. Ther. *26*, 117–127.

34. Glorioso, J.C., Skotte, N.H., Southwell, A.L., Østergaard, M.E., Carroll, J.B., Warby, S.C., Doty, C.N., Petoukhov, E., Vaid, K., Kordasiewicz, H., et al. (2014). Allele-Specific Suppression of Mutant Huntingtin Using Antisense Oligonucleotides: Providing a Therapeutic Option for All Huntington Disease Patients. PLoS One *9*, e107434.

35. Dutrow, E.V., Emera, D., Yim, K., Uebbing, S., Kocher, A.A., Krenzer, M., Nottoli, T., Burkhardt, D.B., Krishnaswamy, S., Louvi, A., and Noonan, J.P. (2022). Modeling uniquely human gene regulatory function via targeted humanization of the mouse genome. Nat. Commun. *13*, 304.

36. Kimi, J., Korczak, P., Vialet, B., Roubin, E., Barthélémy, P., Campagne, S., and Malard, F. (2025). ASOG: AntiSense Oligonucleotide Generator. Comput. Struct. Biotechnol. J. *27*, 4145–4150.

37. Sciabola, S., Xi, H., Cruz, D., Cao, Q., Lawrence, C., Zhang, T., Rotstein, S., Hughes, J.D., Caffrey, D.R., and Stanton, R.V. (2021). PFRED: A computational platform for siRNA and antisense oligonucleotides design. PLoS One *16*, e0238753.

38. Lavorgna, G., Guffanti, A., Borsani, G., Ballabio, A., and Boncinelli, E. (1999). TargetFinder: searching annotated sequence databases for target genes of transcription factors. Bioinformatics *15*, 172–173.

39. Sherry, S.T., Ward, M.H., Kholodov, M., Baker, J., Phan, L., Smigielski, E.M., and Sirotkin, K. (2001). dbSNP: the NCBI database of genetic variation. Nucleic Acids Res. *29*, 308–311.

40. Camacho, C., Coulouris, G., Avagyan, V., Ma, N., Papadopoulos, J., Bealer, K., and Madden, T.L. (2009). BLAST+: architecture and applications. BMC Bioinf. *10*, 421.

41. Lorenz, R., Bernhart, S.H., Höner zu Siederdissen, C., Tafer, H., Flamm, C., Stadler, P.F., and Hofacker, I.L. (2011). ViennaRNA Package 2.0. Algorithm Mol. Biol. *6*, 26.

42. Altschul, S.F., Gish, W., Miller, W., Myers, E.W., and Lipman, D.J. (1990). Basic local alignment search tool. J. Mol. Biol. *215*, 403–410.

43. Lebeau, P.F., Byun, J.H., Platko, K., MacDonald, M.E., Poon, S.V., Faiyaz, M., Seidah, N.G., and Austin, R.C. (2019). Diet-induced hepatic steatosis abrogates cell-surface LDLR by inducing de novo PCSK9 expression in mice. J. Biol. Chem. *294*, 9037–9047.

44. Abifadel, M., Varret, M., Rabès, J.P., Allard, D., Ouguerram, K., Devillers, M., Cruaud, C., Benjannet, S., Wickham, L., Erlich, D., et al. (2003). Mutations in PCSK9 cause autosomal dominant hypercholesterolemia. Nat. Genet. *34*, 154–156.

45. Maxwell, K.N., and Breslow, J.L. (2004). Adenoviral-mediated expression of Pcsk9 in mice results in a low-density lipoprotein receptor knockout phenotype. Proc. Natl. Acad. Sci. USA *101*, 7100–7105.

46. Cohen, J.C., Boerwinkle, E., Mosley, T.H., Jr., and Hobbs, H.H. (2006). Sequence variations in PCSK9, low LDL, and protection against coronary heart disease. N. Engl. J. Med. *354*, 1264–1272.

47. Zhang, Y., Chen, H., Hong, L., Wang, H., Li, B., Zhang, M., Li, J., Yang, L., and Liu, F. (2023). Inclisiran: a new generation of lipid-lowering siRNA therapeutic. Front. Pharmacol. *14*, 1260921.

48. Ray, K.K., Landmesser, U., Leiter, L.A., Kallend, D., Dufour, R., Karakas, M., Hall, T., Troquay, R.P.T., Turner, T., Visseren, F.L.J., et al. (2017). Inclisiran in Patients at High Cardiovascular Risk with Elevated LDL Cholesterol. N. Engl. J. Med. *376*, 1430–1440.

49. Gennemark, P., Walter, K., Clemmensen, N., Rekić, D., Nilsson, C.A.M., Knöchel, J., Hölttä, M., Wernevik, L., Rosengren, B., Kakol-Palm, D., et al. (2021). An oral antisense oligonucleotide for PCSK9 inhibition. Sci. Transl. Med. *13*, eabe9117.

50. Koren, M., Hofherr, A., Schumi, J., Reki, D., Knochel, J., Nilsson, C., Rudvik, A., Wernevik, L., Rydén-Bergsten, T., and Carlsson, B. (2022). Etesian: a phase 2b study of the efficacy, safety and tolerability of azd8233, a pcsk9-targeted antisense oligonucleotide, in patients with dyslipidemia. J. Am. Coll. Cardiol. *79*.

51. Yu, X., and Song, K. (2024). Novel ASO therapeutic target designed against hyperlipidemia via PCSK9 knockdown. Biomed. Pharmacother. *170*, 115960.

52. Sun, X.J., Rothenberg, P., Kahn, C.R., Backer, J.M., Araki, E., Wilden, P.A., Cahill, D.A., Goldstein, B.J., and White, M.F. (1991). Structure of the insulin receptor substrate IRS-1 defines a unique signal transduction protein. Nature *352*, 73–77.

53. Yoneyama, Y., Lanzerstorfer, P., Niwa, H., Umehara, T., Shibano, T., Yokoyama, S., Chida, K., Weghuber, J., Hakuno, F., and Takahashi, S.I. (2018). IRS-1 acts as an endocytic regulator of IGF-I receptor to facilitate sustained IGF signaling. eLife *7*, e32893.

54. Lavin, D.P., White, M.F., and Brazil, D.P. (2016). IRS proteins and diabetic complications. Diabetologia *59*, 2280–2291.

55. Kain, H., Goldblum, D., Geudelin, B., Thorin, E., and Beglinger, C. (2009). Tolerability and safety of GS-101 eye drops, an antisense oligonucleotide to insulin receptor substrate-1: a 'first in man' phase I investigation. Br. J. Clin. Pharmacol. *68*, 169–173.

56. Cursiefen, C., Bock, F., Horn, F.K., Kruse, F.E., Seitz, B., Borderie, V., Früh, B., Thiel, M.A., Wilhelm, F., Geudelin, B., et al. (2009). GS-101 antisense oligonucleotide eye drops inhibit corneal neovascularization: interim results of a randomized phase II trial. Ophthalmology *116*, 1630–1637.

57. Cursiefen, C., Viaud, E., Bock, F., Geudelin, B., Ferry, A., Kadlecová, P., Lévy, M., Al Mahmood, S., Colin, S., Thorin, E., et al. (2014). Aganirsen antisense oligonucleotide eye drops inhibit keratitis-induced corneal neovascularization and reduce need for transplantation: the I-CAN study. Ophthalmology *121*, 1683–1692.

58. Al-Mahmood, S., Colin, S., Farhat, N., Thorin, E., Steverlynck, C., and Chemtob, S. (2009). Potent in vivo antiangiogenic effects of GS-101 (5'-TATCCGGAGGGCT CGCCATGCTGCT-3'), an antisense oligonucleotide preventing the expression of insulin receptor substrate-1. J. Pharmacol. Exp. Therapeut. *329*, 496–504.

59. Qian, Y.Y., Wu, H.Y., Liu, G.Q., Ren, C., Lu, P.R., and Zhang, X.G. (2019). Blockade of insulin receptor substrate-1 inhibits biological behavior of choroidal endothelial cells. Int. J. Ophthalmol. *12*, 1386–1394.

60. Hatcher, J.M., Wu, G., Zeng, C., Zhu, J., Meng, F., Patel, S., Wang, W., Ficarro, S.B., Leggett, A.L., Powell, C.E., et al. (2018). SRPKIN-1: A Covalent SRPK1/2 Inhibitor that Potently Converts VEGF from Pro-angiogenic to Anti-angiogenic Isoform. Cell Chem. Biol. *25*, 460–470.e6.

61. Gammons, M.V.R., Dick, A.D., Harper, S.J., and Bates, D.O. (2013). SRPK1 inhibition modulates VEGF splicing to reduce pathological neovascularization in a rat model of retinopathy of prematurity. Investig. Ophthalmol. Vis. Sci. *54*, 5797–5806.

62. Targher, G., Valenti, L., and Byrne, C.D. (2025). Metabolic Dysfunction–Associated Steatotic Liver Disease. N. Engl. J. Med. *393*, 683–698.

63. Krahmer, N., Walther, T.C., and Farese, R.V., Jr. (2025). The pathogenesis of hepatic steatosis in MASLD: a lipid droplet perspective. J. Clin. Investig. *135*, e198334.

64. Duan, J., Wang, Z., Duan, R., Yang, C., Zhao, R., Feng, Q., Qin, Y., Jiang, J., Gu, S., Lv, K., et al. (2022). Therapeutic targeting of hepatic ACSL4 ameliorates NASH in mice. Hepatology *75*, 140–153.

65. Li, X., Chen, Q., Zhao, D., Tan, J., Liao, R., Gu, Y., Zhu, J., Zhang, H., Xie, J., and Chen, L. (2025). ACSL4 accelerates osteosarcoma progression via modulating TGF-β/Smad2 signaling pathway. Mol. Cell. Biochem. *480*, 549–562.

66. Crunkhorn, S. (2025). A platform for personalized ASO therapeutics. Nat. Rev. Drug Discov. *24*, 249.

67. Østergaard, M.E., Southwell, A.L., Kordasiewicz, H., Watt, A.T., Skotte, N.H., Doty, C.N., Vaid, K., Villanueva, E.B., Swayze, E.E., Bennett, C.F., et al. (2013). Rational design of antisense oligonucleotides targeting single nucleotide polymorphisms for potent and allele selective suppression of mutant Huntingtin in the CNS. Nucleic Acids Res. *41*, 9634–9650.

68. Jackson, A.L., Bartz, S.R., Schelter, J., Kobayashi, S.V., Burchard, J., Mao, M., Li, B., Cavet, G., and Linsley, P.S. (2003). Expression profiling reveals off-target gene regulation by RNAi. Nat. Biotechnol. *21*, 635–637.

69. Burdick, A.D., Sciabola, S., Mantena, S.R., Hollingshead, B.D., Stanton, R., Warneke, J.A., Zeng, M., Martsen, E., Medvedev, A., Makarov, S.S., et al. (2014). Sequence motifs associated with hepatotoxicity of locked nucleic acid—modified antisense oligonucleotides. Nucleic Acids Res. *42*, 4882–4891.

70. Velliou, R.I., Giannousi, E., Ralliou, C., Kassi, E., and Chatzigeorgiou, A. (2024). *Ex Vivo* Tools and Models in MASLD Research. Cells *13*.

71. Dobin, A., Davis, C.A., Schlesinger, F., Drenkow, J., Zaleski, C., Jha, S., Batut, P., Chaisson, M., and Gingeras, T.R. (2013). STAR: ultrafast universal RNA-seq aligner. Bioinformatics *29*, 15–21.

72. Liao, Y., Smyth, G.K., and Shi, W. (2014). featureCounts: an efficient general purpose program for assigning sequence reads to genomic features. Bioinformatics *30*, 923–930.

73. Love, M.I., Huber, W., and Anders, S. (2014). Moderated estimation of fold change and dispersion for RNA-seq data with DESeq2. Genome Biol. *15*, 550.

74. Wu, T., Hu, E., Xu, S., Chen, M., Guo, P., Dai, Z., Feng, T., Zhou, L., Tang, W., Zhan, L., et al. (2021). clusterProfiler 4.0: A universal enrichment tool for interpreting omics data. Innovation *2*, 100141.

OMTN, Volume *37*

## Supplemental information

# ClinASO: An open-source platform for rapid drug discovery of gapmer antisense oligonucleotides

**Shunkai Chen, Hao Liu, Dezi Cong, Ao Dong, Yuhang Wang, Jingyi Bi, Shijie Guo, Juan Yang, Xiaolei Wang, Guiping Ren, Ke Zhang, Haisheng Wang, Fan Lai, and Yunkun Dang**

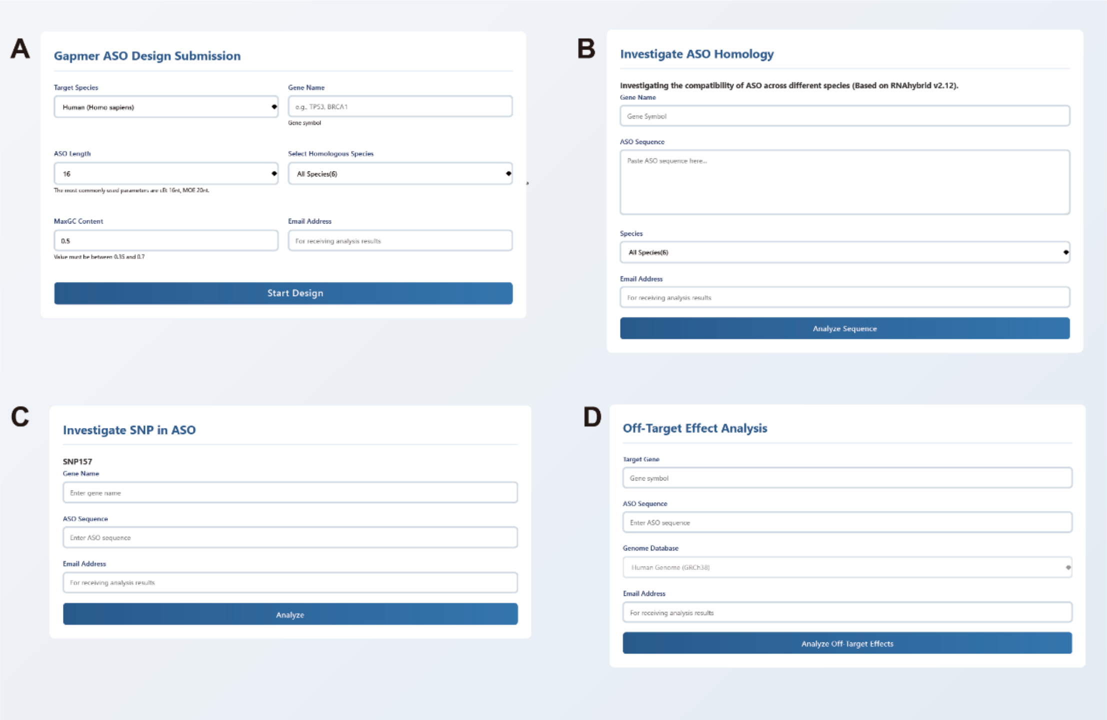


**Figure S1. Overview of the four main functional interfaces of the ClinASO platform.**

(A) Gapmer ASO design interface.

(B) ASO homology analysis interface.

(C) SNP Investigate tool for ASOs.

(D) Off-target prediction interface

**A**

| Drug Name | Trade Name | Developer | Approval Year | Target |
|---|---|---|---|---|
| Fomivirsen | Vitravene | Ionis Pharmaceuticals/Novartis | 1998 | CMV |
| Mipomersen | Kynamro | Ionis/Sanofi | 2013 | ApoB-100 |
| Inotersen | Tegsedi | Ionis/Akcea Therapeutics | 2018 | TTR |
| Tofersen | Qalsody | Ionis/Biogen | 2023 | SOD1 |
| Eplontersen | Wainua | Ionis/AstraZeneca | 2023 | TTR |
| Olezarsen | Tryngolza | Ionis Pharmaceuticals | 2024 | APOC3 |
| Donidalorsen | Dawnzera | Ionis Pharmaceuticals | 2025 | KLKB1 |

**B**

| Target | ID | chr | start | end | ASO sequence | RNase H score | GC content | ASO MFE | 4 mismatch genes | 3 mismatch genes | 2 mismatch genes |
|---|---|---|---|---|---|---|---|---|---|---|---|
| SOD1 | Tofersen | NC_000021.9 | 31668629 | 31668649 | CAGGATACATTTCTACAGCT | -0.316312356 | 0.4 | -0.8 | 0 | 0 | 0 |
| APOB100 | Mipomersen | NC_000002.12 | 21016630 | 21016650 | GCCTCAGTCTGCTTCGCACC | -0.438978448 | 0.65 | -0.3 | 0 | 0 | 0 |
| KLKB1 | Donidalorsen | NC_000004.12 | 186251565 | 186251585 | TGCAAGTCTCTTGGCAAACA | -0.345786073 | 0.45 | -2 | 0 | 0 | 1 |
| TTR | Inotersen | NC_000018.10 | 31598713 | 31598733 | CTCTTGGTTACATGAAATCC | 0.403416403 | 0.4 | -1.1 | 1 | 0 | 0 |
| APOC3 | ASO2694 | NC_000011.10 | 116833045 | 116833065 | AGCTTCTTGTCCAGCTTTAT | -0.583327284 | 0.4 | -1.8 | 0 | 0 | 0 |
| GFAP | zilganersen | NC_000017.11 | 44909448 | 44909468 | CAGTATTACCTCTACTAGTC | -0.164973392 | 0.4 | -0.2 | 0 | 0 | 0 |
| FUS | Ulefnersen | NC_000016.10 | 31186181 | 31186201 | GCAATGTCACCTTTCATACC | 0.224329296 | 0.45 | 0 | 0 | 0 | 0 |
| DGAT2 | ION224 | NC_000011.10 | 75785888 | 75785908 | TGCCATTTAATGAGCTTCAC | -0.014613995 | 0.4 | -1.4 | 0 | 0 | 0 |
| PCSK9 | AZD8233 | NC_000001.11 | 55042353 | 55042369 | TAATCTCATGTCAGTT | -0.615086151 | 0.3125 | 0 | 1 | 0 | 0 |

| Target | ID | 1 mismatch genes | 0 mismatch genes | mismatch genes name | ASO position | Crab-eating macaque | Mouse | Rat | Pig | Rabbit | Guinea pig |
|---|---|---|---|---|---|---|---|---|---|---|---|
| SOD1 | Tofersen | 1 | 0 | PRDM1 | intron | 0.95 | 0.65 | 0.7 | 0.6 | 0.8 | 0.6 |
| APOB100 | Mipomersen | 0 | 0 | N/A | intron | 0.90 | 0.75 | 0.70 | 0.65 | 0.90 | 0.65 |
| KLKB1 | Donidalorsen | 1 | 0 | CDH4/GABRG3 | exon | 0.95 | 0.9 | 0.85 | 0.95 | 0.85 | 0.95 |
| TTR | Inotersen | 0 | 0 | LOC124904277 | intron | 1 | 0.65 | 0.6 | 0.8 | 0.8 | 0.8 |
| APOC3 | ASO2694 | 3 | 0 | CACNA1C/CACNA1C/NEURL1 | intron | 1.00 | 0.95 | 0.95 | 0.85 | 0.90 | |
| GFAP | zilganersen | 0 | 0 | N/A | intron | 0.95 | 0.7 | 0.65 | 0.65 | 0.65 | 0.65 |
| FUS | Ulefnersen | 2 | 0 | BTRC/ERP44 | intron | 1 | 1 | 1 | 1 | 1 | 1 |
| DGAT2 | ION224 | 3 | 0 | LOC124900690/CAND1.11/RBP | intron | 1 | 0.75 | 0.7 | 0.75 | 0.8 | 0.75 |
| PCSK9 | AZD8233 | 0 | 0 | LATS1 | intron | 1 | 0.75 | 0.75 | 0.69 | 0.62 | |

**Figure S2. Parameter information of FDA approved ASOs and those in clinical trials within the ClinASO.**

(A) FDA approved gapmer ASOs.

(B) Parameter information of ASOs in clinical trials within ClinASO.

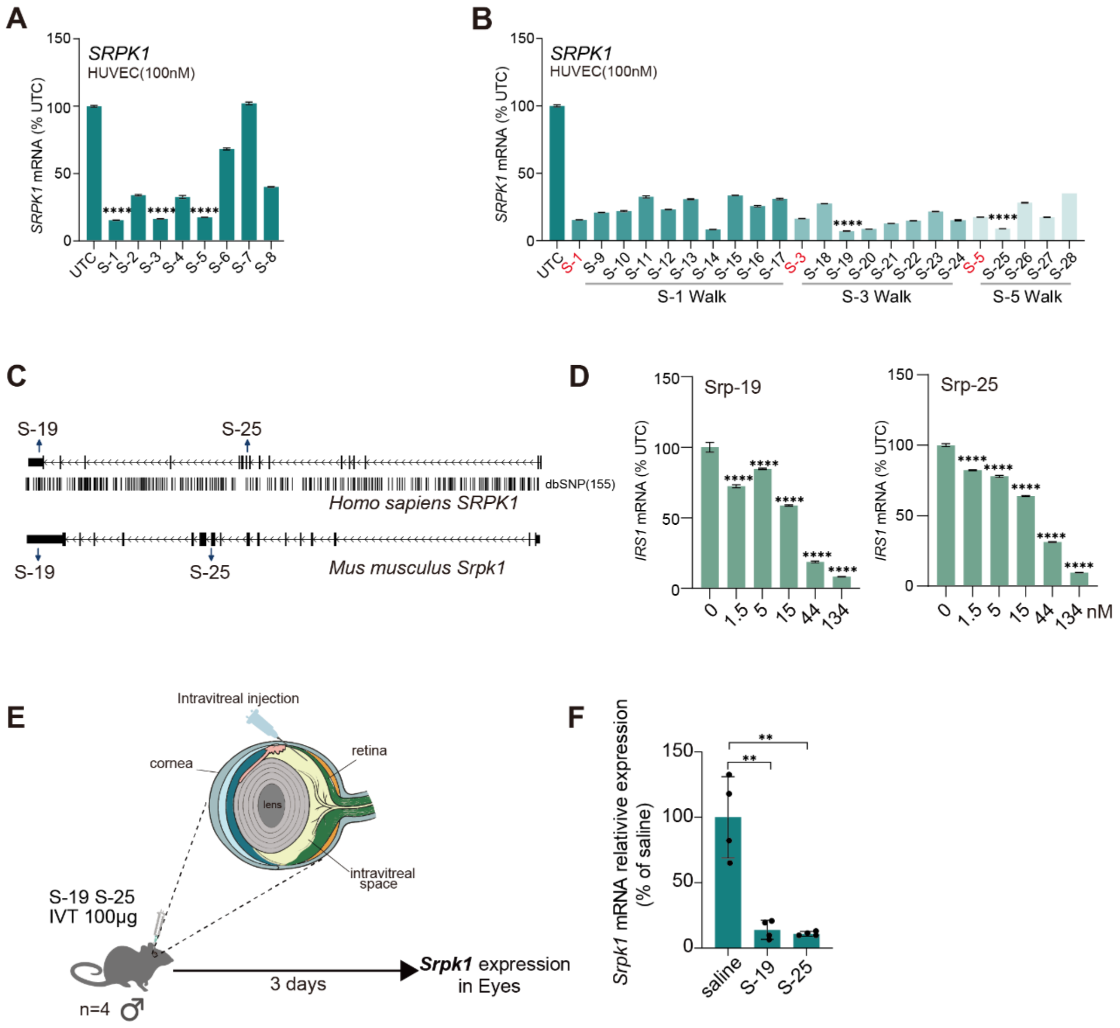


**Figure S3. Efficacy of ASOs targeting *SRPK1*.**

(A & B) Barplots showing preliminary screening and hotspot identification of ASOs targeting *SRPK1* in HUVECs transfected at 100nM for 24h, measured by RT-qPCR with *β-Actin* as internal control.

(C) Schematic representation of the targeting sites of candidate ASOs (S-19 and S-25) on the homologous *SRPK1* sequence.

(D) Barplots showing quantification of *SRPK1* mRNA knockdown by RT-qPCR with *β-Actin* as internal control in HUVECs 24 h after transfection with increasing concentrations of ASOs. Data are mean ± SD; *$p < 0.05$, **$p < 0.01$, ***$p < 0.001$, ****$p < 0.0001$ versus control (one-way ANOVA).

(E) Schematic representation of intravitreal injection in mice for evaluating *SRPK1*-targeting ASOs.

(F) Barplots showing *Srpk1* mRNA levels in mouse ocular tissues three days after intravitreal administration of ASOs S-19 or S-25 (100μg per eye), quantified by RT-qPCR with *β-Actin* as internal control. Data are mean ± SD (n = 4 biological replicates)**.** $p < 0.01$ versus saline-treated controls (one-way ANOVA).

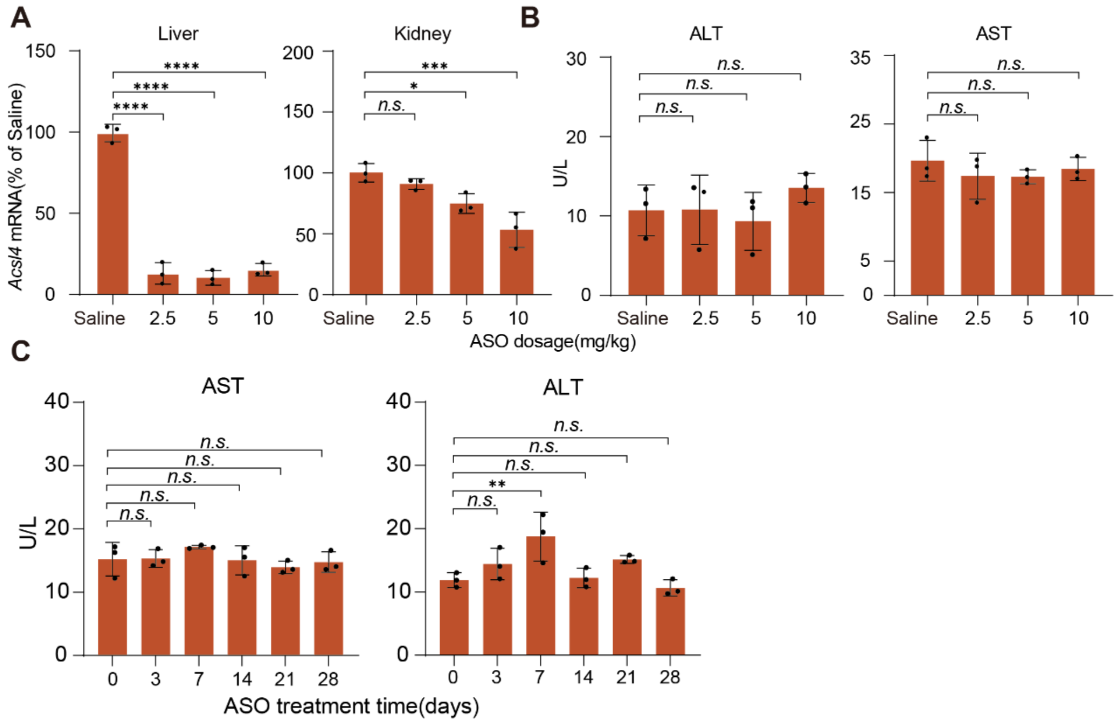


**Figure S4. Evaluation of the knockdown efficiency and safety of GA-28c**

(A) Barplots showing *ACSL4* mRNA levels in liver and kidney tissues from the experiment shown in (C), quantified by RT-qPCR with *GAPDH* as internal control. Data are presented as mean ± SD; n = 3 biological replicates.

(B) Barplots showing Serum alanine aminotransferase (ALT) and aspartate aminotransferase (AST) levels in mice from the experiment shown in (C). Data are presented as mean ± SD; n = 3 biological replicates.

(C) Barplots showing Serum ALT and AST levels measured at the indicated time points during the treatment of GA-28c. Data are presented as mean ± SD; n = 3 biological replicates.

For panel A, B and C, Statistical significance was determined by one-way ANOVA: *n.s.*: no significance, *$p < 0.05$, **$p < 0.01$, ***$p < 0.001$, ****$p < 0.0001$.

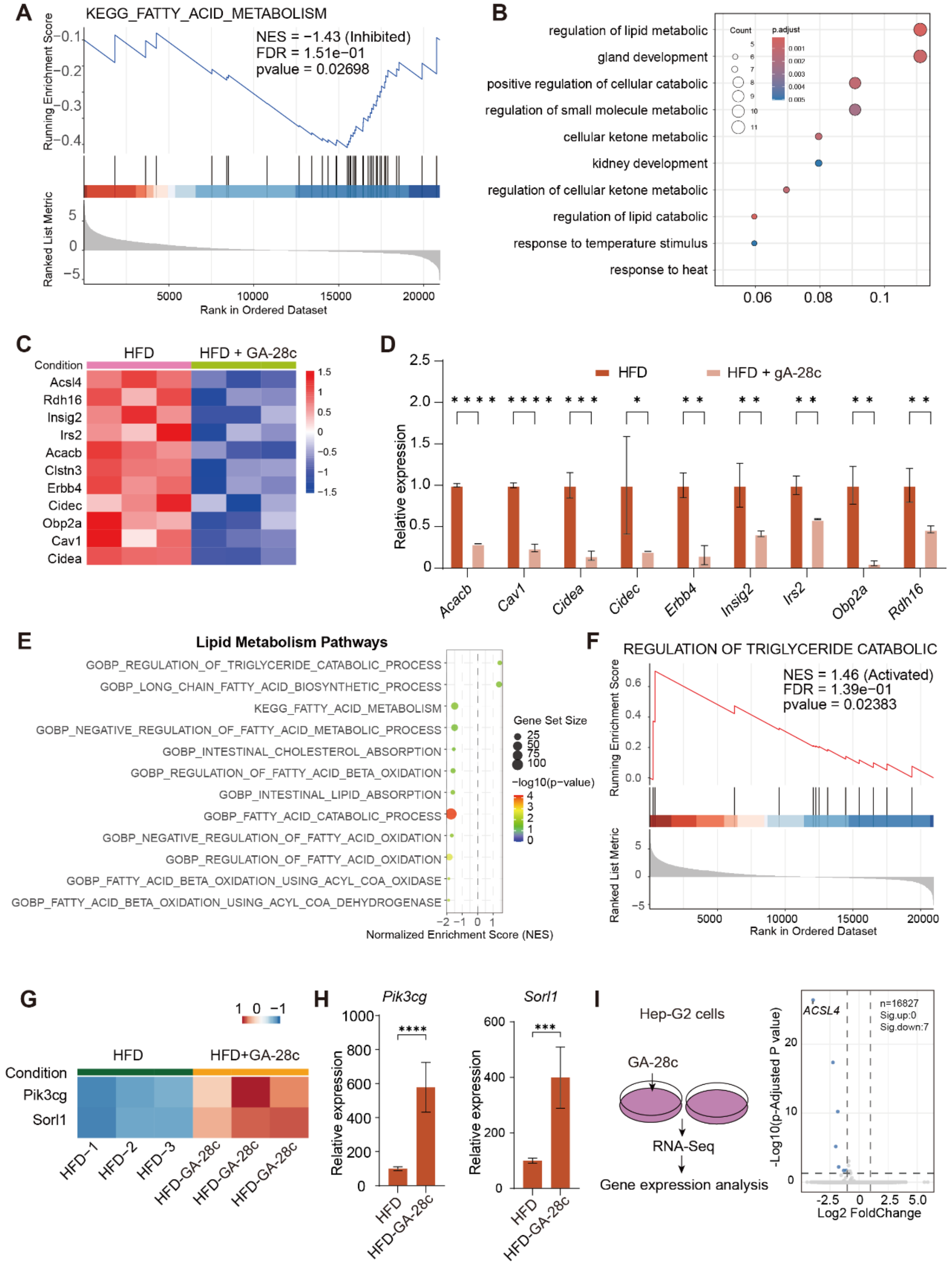


**Figure S5. Mechanistic analyses of therapeutic effects of GA-28c in HFD mice**

(A) Gene-set enrichment analysis (GSEA) of RNA-seq data (n = 3 biological replicates per group) on KEGG Fatty acid metabolism pathway in HFD and GA-28c-treated mouse livers vs HFD control (NES = –1.43).

(B) Gene Ontology (GO) enrichment map of the down-regulated genes GA-28c-treated mouse livers vs HFD control.

(C) Heatmap showing gene expression profiles involved in lipid-metabolism of 3 individual mice.

(D) Barplots showing RT-qPCR with *GAPDH* as internal control validation of expression 9 representative genes from panel C. Data are presented as mean ± SD; n = 3. Student's *t* test, * $p < 0.05$, ** $p < 0.01$, *** $p < 0.001$, **** $p < 0.0001$.

(E) Bubble plot summarizing GSEA of all lipid-related KEGG pathways for RNA-seq of HFD and GA-28c-treated vs HFD mice.

(F) GSEA analysis for the GO term Regulation of triglyceride catabolic process for livers of HFD and GA-28c-treated vs HFD mice.

(G) Heat-map of the *Pik3cg* and *Sorl1* expression from (F).

(H) Barplots showing RT-qPCR results of *Pik3cg* and *Sorl1* induction with *GAPDH* as internal control. Bars represent fold-change. Data are presented as mean ± SD; n = 3. Student's *t* test, ***$p < 0.001$, ****$p < 0.0001$.

(I) Cartoon showing Off-target effect of GA-28c (44nM) in HepG2 cells and volcano plot of RNA-seq. (n = 3, |log2FC| > 1 and *p* adjust value < 0.05)).

**Table S1: Information on target-specific antisense oligonucleotides against *ACSL4, SRPK1, IRS1,* and *PCSK9*.** Due to its length, this supplemental table cannot fit within two 8.5" × 11" pages and has therefore been uploaded as a separate Excel file.

## Table S2: Sequences of antisense oligonucleotides designed for ACSL4 and SRPK1(Walk)

| Gene | ACSL4 | SRPK1 | |
|---|---|---|---|
| A-19 Walk | | S-1 Walk | |
| ID | Sequence | ID | Sequence |
| A-22 | GCGTTTTTCTTCTTGGCATT | S-9 | TGCCACAATGAGCTTGCGAG |
| A-23 | AGCGTTTTTCTTCTTGGCAT | S-10 | GCCACAATGAGCTTGCGAGG |
| A-24 | TAGCGTTTTTCTTCTTGGCA | S-11 | CCACAATGAGCTTGCGAGGC |
| A-25 | ATAGCGTTTTTCTTCTTGGC | S-12 | CACAATGAGCTTGCGAGGCA |
| A-26 | CATAGCGTTTTTCTTCTTGG | S-13 | CAATGAGCTTGCGAGGCACC |
| A-19 | CCATAGCGTTTTTCTTCTTG | S-14 | AATGAGCTTGCGAGGCACCT |
| A-27 | GCCATAGCGTTTTTCTTCTT | S-15 | ATGAGCTTGCGAGGCACCTT |
| A-28 | TGCCATAGCGTTTTTCTTCT | S-16 | TGAGCTTGCGAGGCACCTTC |
| A-29 | TTGCCATAGCGTTTTTCTTC | S-17 | GAGCTTGCGAGGCACCTTCC |
| A-30 | TTTGCCATAGCGTTTTTCTT | S-3 Walk | |
| A-31 | CTTTGCCATAGCGTTTTTCT | ID | Sequence |
| A-5 Walk | | S-18 | CCGCGGGATTCAGCGCCGAC |
| ID | Sequence | S-19 | TCCGCGGGATTCAGCGCCGA |
| A-32 | TGGCCATGTTTAAGATTCTT | S-20 | GTCCGCGGGATTCAGCGCCG |
| A-33 | GGCCATGTTTAAGATTCTTC | S-21 | CGTCCGCGGGATTCAGCGCC |
| A-5 | GCCATGTTTAAGATTCTTCT | S-22 | TCGTCCGCGGGATTCAGCGC |
| A-34 | CCATGTTTAAGATTCTTCTA | S-23 | GTCGTCCGCGGGATTCAGCG |
| A-35 | CATGTTTAAGATTCTTCTAA | S-24 | GGGTCGTCCGCGGGATTCAG |
| A-36 | ATGTTTAAGATTCTTCTAAT | S-5 Walk | |
| A-37 | TGTTTAAGATTCTTCTAATG | ID | Sequence |
| A-38 | GTTTAAGATTCTTCTAATGC | S-25 | CTGCATTCGCTTCTCTAGTA |
| A-39 | TTTAAGATTCTTCTAATGCA | S-26 | GCATTCGCTTCTCTAGTAAT |
| | | S-27 | CATTCGCTTCTCTAGTAATT |
| | | S-28 | ATTCGCTTCTCTAGTAATTC |

**Table S3**: ***PCSK9 ACSL4* Low-Efficiency Score ASOs.** Due to its length, this supplemental table cannot fit within two 8.5" × 11" pages and has therefore been uploaded as a separate Excel file.
